# Two Genes on A/J Chromosome 18 Are Associated with Susceptibility to *Staphylococcus aureus* Infection by Combined Microarray and QTL Analyses

**DOI:** 10.1371/journal.ppat.1001088

**Published:** 2010-09-02

**Authors:** Sun-Hee Ahn, Hitesh Deshmukh, Nicole Johnson, Lindsay G. Cowell, Thomas H. Rude, William K. Scott, Charlotte L. Nelson, Aimee K. Zaas, Douglas A. Marchuk, Sehoon Keum, Supaporn Lamlertthon, Batu K. Sharma-Kuinkel, Gregory D. Sempowski, Vance G. Fowler

**Affiliations:** 1 Department of Medicine, Duke University Medical Center, Durham, North Carolina, United States of America; 2 Department of Biostatistics and Bioinformatics, Duke University Medical Center, Durham, North Carolina, United States of America; 3 Dr. John T. Macdonald Foundation Department of Human Genetics and John P. Hussman Institute for Human Genomics, Miller School of Medicine, University of Miami, Miami, Florida, United States of America; 4 Duke Clinical Research Institute, Durham, North Carolina, United States of America; 5 Department of Molecular Genetics and Microbiology, Duke University Medical Center, Durham, North Carolina, United States of America; 6 Duke Human Vaccine Institute, Durham, North Carolina, United States of America; Children's Hospital Boston, United States of America

## Abstract

Although it has recently been shown that A/J mice are highly susceptible to *Staphylococcus aureus* sepsis as compared to C57BL/6J, the specific genes responsible for this differential phenotype are unknown. Using chromosome substitution strains (CSS), we found that loci on chromosomes 8, 11, and 18 influence susceptibility to *S. aureus* sepsis in A/J mice. We then used two candidate gene selection strategies to identify genes on these three chromosomes associated with *S. aureus* susceptibility, and targeted genes identified by both gene selection strategies. First, we used whole genome transcription profiling to identify 191 (56 on chr. 8, 100 on chr. 11, and 35 on chr. 18) genes on our three chromosomes of interest that are differentially expressed between *S. aureus*-infected A/J and C57BL/6J. Second, we identified two significant quantitative trait loci (QTL) for survival post-infection on chr. 18 using N_2_ backcross mice (F_1_ [C18A]×C57BL/6J). Ten genes on chr. 18 (*March3*, *Cep120*, *Chmp1b*, *Dcp2*, *Dtwd2*, *Isoc1*, *Lman1*, *Spire1*, *Tnfaip8*, and *Seh1l*) mapped to the two significant QTL regions and were also identified by the expression array selection strategy. Using real-time PCR, 6 of these 10 genes (*Chmp1b*, *Dtwd2*, *Isoc1*, *Lman1*, *Tnfaip8*, and *Seh1l*) showed significantly different expression levels between *S. aureus*-infected A/J and C57BL/6J. For two (*Tnfaip8 and Seh1l*) of these 6 genes, siRNA-mediated knockdown of gene expression in *S. aureus*–challenged RAW264.7 macrophages induced significant changes in the cytokine response (IL-1 β and GM-CSF) compared to negative controls. These cytokine response changes were consistent with those seen in *S. aureus*-challenged peritoneal macrophages from CSS 18 mice (which contain A/J chromosome 18 but are otherwise C57BL/6J), but not C57BL/6J mice. These findings suggest that two genes, *Tnfaip8 and Seh1l*, may contribute to susceptibility to *S. aureus* in A/J mice, and represent promising candidates for human genetic susceptibility studies.

## Author Summary

*Staphylococcus aureus* has a wide spectrum of human infection, ranging from asymptomatic nasal carriage to overwhelming sepsis and death. Mouse models offer an attractive strategy for investigating complex diseases such as *S. aureus* infections. A/J mice are highly susceptible to *S. aureus* infection compared with C57BL/6J mice. We showed that genes on chromosomes 8, 11, and 18 in A/J are responsible for susceptibility to *S. aureus* by using chromosome substitution strains (CSS). From the ∼4200 genes on these three chromosomes, we identified 191 which were differentially expressed between A/J and C57BL/6J when challenged with *S. aureus*. Next, we identified two significant QTLs on chromosome 18 that are associated with susceptibility to *S. aureus* infection in N_2_ backcross mice. Ten genes (*March3*, *Cep120*, *Chmp1b*, *Dcp2*, *Dtwd2*, *Isoc1*, *Lman1*, *Spire1*, *Tnfaip8*, and *Seh1l*) mapped to the two significant QTLs and were differentially expressed between A/J and C57BL/6J. One gene on each QTL, *Tnfaip8* and *Seh1l*, affected expression of cytokines in mouse macrophages exposed to *S. aureus*. These cytokine response patterns were consistent with those seen in *S. aureus*-challenged peritoneal macrophages from CSS 18, but not C57BL/6J. *Tnfaip8* and *Seh1l* are strong candidates for genes influencing susceptibility to *S. aureus* of A/J mice.

## Introduction

*Staphylococcus aureus* is an important human pathogen whose clinical spectrum ranges from asymptomatic colonization to endocarditis, shock, and death. Although the importance of genetic factors in determining host susceptibility to *S. aureus* colonization and infection [1]–[3] is generally accepted, the specific genes responsible for this susceptibility are largely unknown. As with most infectious diseases, the genetics of host susceptibility to *S. aureus* is complex, resulting from variation in multiple genes of small to moderate effect, and from the interaction of these genes with non-genetic factors [4].

Mouse models offer an attractive strategy for investigating complex diseases such as *S. aureus* infections. Abundant breeding, short gestation periods, and the availability of extensive sequence data for inbred strains (http://mouse.perlegen.com/mouse/index.html and http://www.informatics.jax.org/) facilitate genetic research in mice. In addition, inbred mouse strains demonstrate considerable strain variation in susceptibility to various pathogens [5]–[11], including *S. aureus* [3]. The determinants of this differential susceptibility to pathogens are probably multigenic, and largely unknown [12].

Among the inbred mouse strains, A/J was recently shown to be highly susceptible to *S. aureus* infection compared with C57BL/6J [3]. By chance, these same genetic backgrounds (A/J and C57BL/6J) were used to construct murine chromosome substitution strains (CSS). CSS were developed to speed the genetic mapping of heritable traits, and were created from a host C57BL/6J strain and a donor A/J inbred strain [13]. Each CSS mouse is homosomic for a specified A/J chromosome but otherwise has a C57BL/6J background. Breeding strategies using these CSS strains greatly enhance the detection of quantitative trait loci (QTLs). Using CSS mice, the impact of a single A/J chromosome can be effectively isolated by eliminating the contribution to variance in phenotype from the other A/J chromosomes. CSS strains have been used to identify QTL for several complex traits, including anxiety [14], diet-induced obesity [15] serum levels of sterols and amino acids [15], testicular cancer [16], [17], pubertal timing [18], airway hyperresponsiveness [19], and seizures [20], and provide an ideal means of identifying genetic determinants of murine susceptibility to *S. aureus* sepsis.

**Figure 1:**
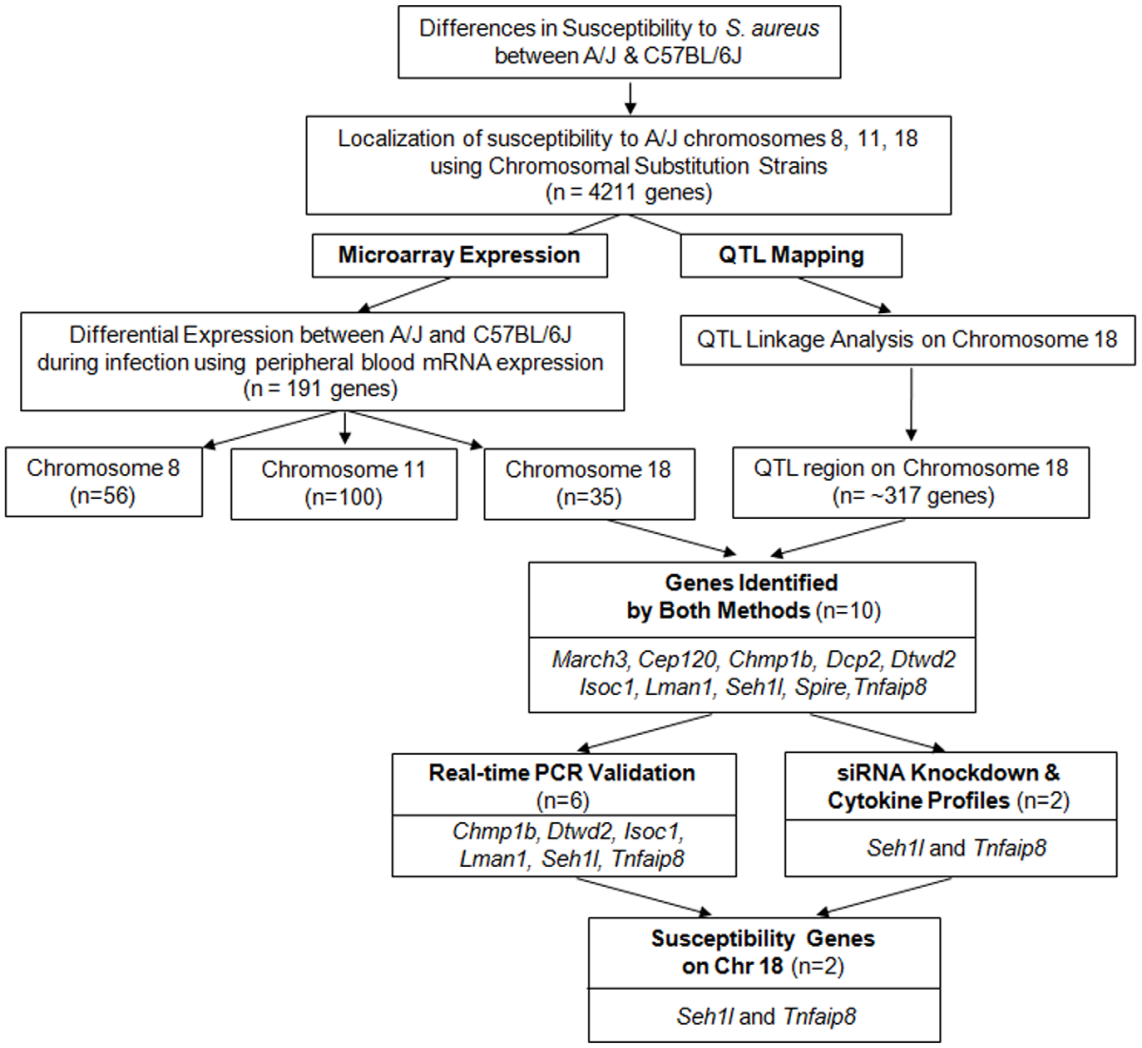
Overall strategy for identifying genes associated with susceptibility to *S. aureus* in A/J mice.

In this study, we sought to identify genetic factors associated with susceptibility to *S. aureus* using a multi-step selection process (Figure 1). First, we identified individual chromosomes governing susceptibility to *S. aureus* infection in A/J mice by phenotyping a complete panel of CSS mice. Next, we used peripheral blood mRNA expression to identify genes on these chromosomes that were differentially expressed between susceptible (A/J) and resistant (C57BL/6J) mouse strains in the setting of *S. aureus* infection. Following this, we identified two QTL regions on chromosome 18 that are significantly associated with susceptibility to infection with *S. aureus*. From these two QTL regions, we identified 10 candidate genes that were differentially expressed between A/J and C57BL/6J. We found evidence of biological relevance for two of these genes (*Tnfaip8* and *Seh1l*) using siRNA mediated knockdown and Luminex-based cytokine profiles in *S. aureus*-challenged RAW264.7 mouse macrophages, as well as peritoneal macrophages from CSS18 mice.

## Results

### Differential susceptibility to *S. aureus* in A/J and C57BL/6J

**Figure 2:**
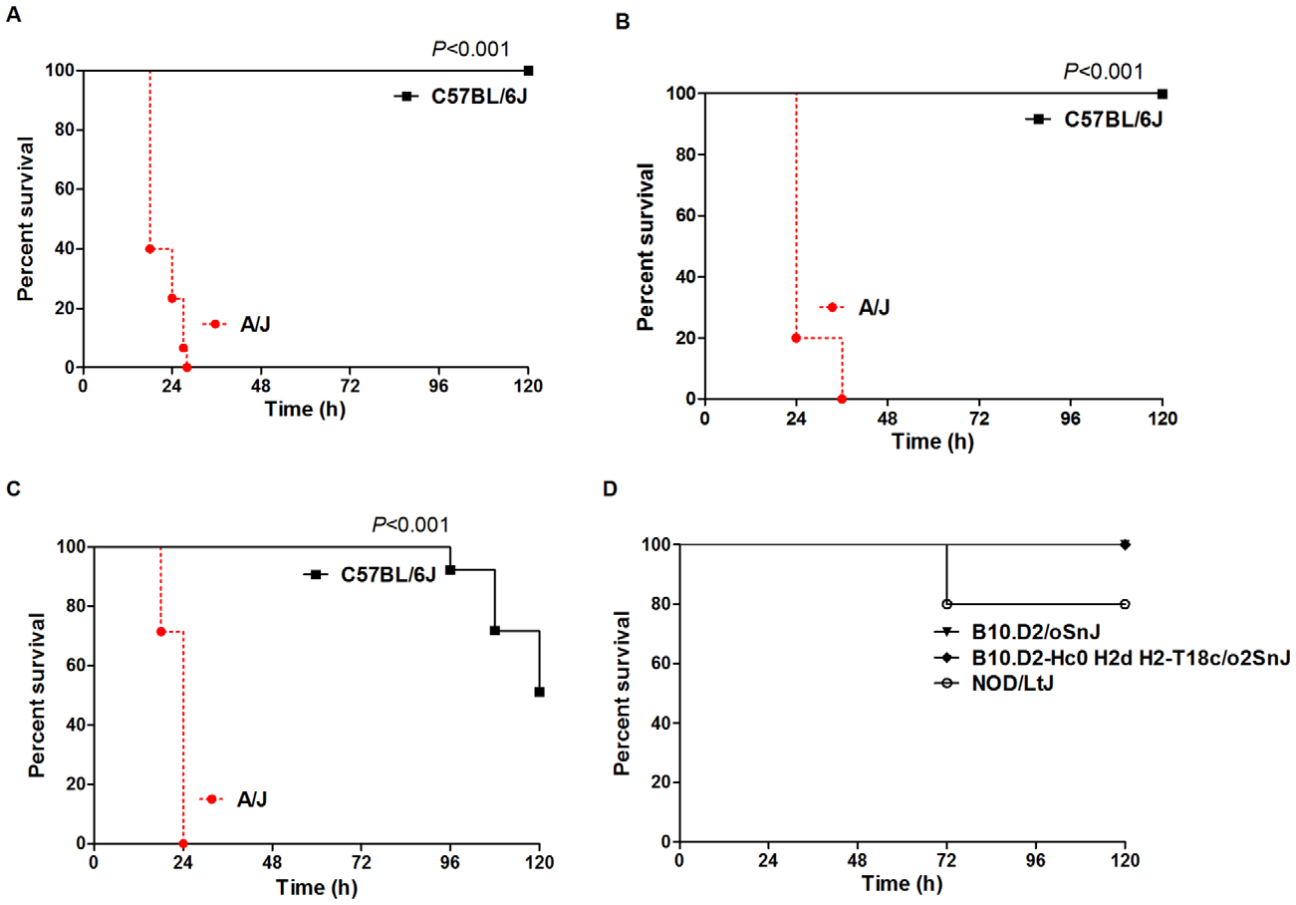
A/J and C57BL/6J mice exhibit different susceptibility to *S. aureus* that is not primarily due to deficiency in Complement C5. C57BL/6J and A/J were injected via intraperitoneal (i.p.) route (10^7^ CFU/g) with *S. aureus* strains Sanger 476 (Figure 2A) and MW2 (Figure 2B) (n = 15 mice for each strain). (Figure 2C) C57BL/6J and A/J mice were injected via tail vein route (10^7^ CFU/g) with Sanger 476 (n = 10 mice for each strain). Comparison of survival curves was performed by use of log rank test. Values of *P*<0.05 were considered significant. (Figure 2D) Deficiency of Complement C5 is not the primary determinant of susceptibility to *S. aureus* in A/J mice. Three C5-deficient mouse strains (C57BL/10SnJ, B10.D2/oSnJ, B10.D2-Hc0 H2d H2-T18c/o2SnJ, and NOD/LtJ) were injected i.p. (10^7^ CFU/g) with Sanger 476 (n = 10 mice for each strain).

When mice were injected (intraperitoneally [i.p.]) with the Sanger 476 strain of *S. aureus*, C57BL/6J mice demonstrated a resistant phenotype (median survival >120 h), whereas A/J mice demonstrated a susceptible phenotype (median survival: 22 h) (Figure 2A). This pattern of differential susceptibility to *S. aureus* between C57BL/6J and A/J mice persisted when the experiments were repeated using both a different *S. aureus* strain (MW2, a methicillin resistant *S. aureus* [MRSA] isolate) (Figure 2B), and when mice were infected by intravenous rather than i.p. route (Figure 2C). A/J mice are known to be deficient in complement factor C5, an important component in neutrophil and macrophage recruitment [21]–[23]. Thus, the potential impact of C5 deficiency on susceptibility to *S. aureus* infection is important to consider. To ensure that A/J's susceptibility to *S. aureus* is not primarily due to C5 deficiency, we challenged three additional C5 deficient mouse strains (B10.D2/oSnJ, B10.D2-Hc0 H2d H2-T18c/o2SnJ, and NOD/LtJ) with an intraperitoneal injection of *S. aureus* (10^7^ CFU/g). All three additional C5-deficient mice were resistant to *S. aureus* infection (median survival >120 h, Figure 2D), suggesting that factors other than C5 deficiency were responsible for the susceptibility of A/J mice to *S. aureus*.

### Genetic susceptibility to *S. aureus* in mice localizes to A/J chromosomes 8, 11, and 18

Since we identified C57BL/6J mice as resistant and A/J mice as susceptible to *S. aureus* infection, we used CSS from these backgrounds to further localize the chromosomal regions responsible for these divergent phenotypes. CSS mice with chromosome 8, 11 or 18 from the susceptible background (A/J) transferred individually into the otherwise resistant background (C57BL/6J) were significantly more susceptible to *S. aureus* infection compared to C57BL/6J mice (*p*<0.005, log rank test) (**Table 1**) or other chromosome substituted strains. This suggests that genetic loci mediating susceptibility to *S. aureus* are located on each of these 3 chromosomes.

**Table 1:** Median survival of C57BL/6J, A/J, and Chromosome substituted strain (CSS) mice after intraperitoneal injection with *S. aureus* (10^7^ CFU/g).

Strain	Median Survival (d)	Number of Mice Tested (n)	*P*-Value*
**A/J**	1	10	0.0005
**C57BL/6J**	>5	10	1
**CSS 1**	>5	10	1
**CSS 2**	>5	10	1
**CSS 3**	>5	10	1
**CSS 4**	>5	10	1
**CSS 5**	>5	10	1
**CSS 6**	>5	10	1
**CSS 7**	2.5	10	0.0771
**CSS 8**	1.5	10	0.0027
**CSS 9**	>5	10	1
**CSS 10**	>5	10	1
**CSS 11**	1	10	0.0023
**CSS 12**	>5	10	1
**CSS 13**	>5	10	1
**CSS 14**	>5	10	1
**CSS 15**	>5	10	1
**CSS 16**	>5	10	1
**CSS 17**	>5	10	1
**CSS 18**	1	10	0.0023
**CSS 19**	>5	10	1
**CSS X**	4	5	0.0805
**CSS Y**	>5	5	1

*CSS mice contain a single A/J chromosome inserted in C57BL/6J background. Thus, CSS 1 contains A/J chromosome 1 inserted in C57BL/6J background, etc. CSS strains 8, 11 and 18 were significantly more susceptible to S. aureus infection than C57BL/6J (P = 0.0027, 0.0023, and 0.0023, respectively).*

*Compared to survival for C57BL/6J. Comparison of survival curves was performed by use of log rank test. Values of P<0.05 were considered significant.

### Rapid mortality in CSS mice with A/J “susceptibility” chromosomes 8, 11, and 18 is associated with increased bacterial load in the peritoneal fluid and kidneys

**Figure 3:**
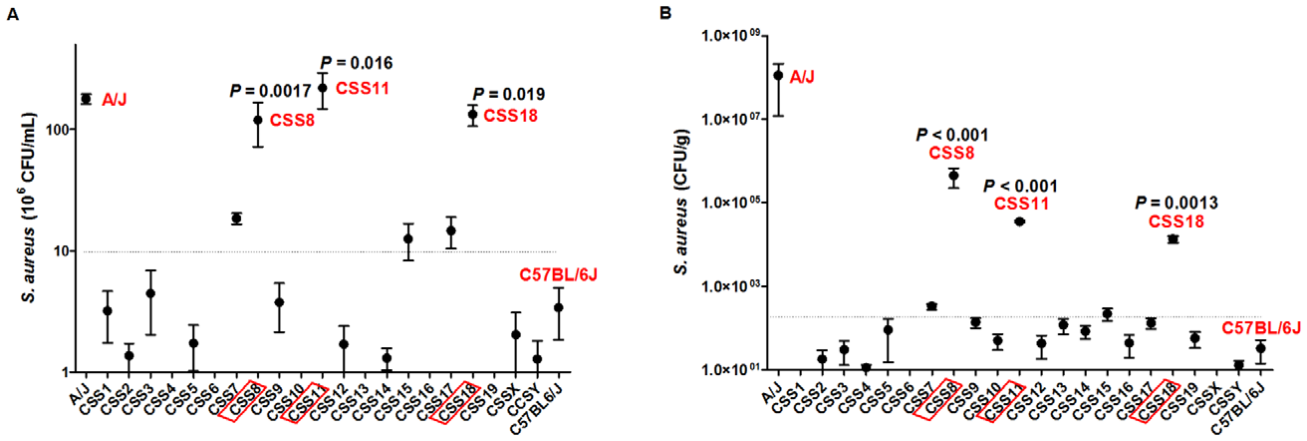
Bacterial load in peritoneal fluid and kidneys is higher in chromosome substituted strain (CSS) 8, CSS11 and CSS18 mice after *S. aureus* injection. (A) Bacterial load in peritoneal fluid. C57BL/6J, A/J, and CSS mice were injected (i.p.) with Sanger 476 (10^7^ CFU/g) and euthanized 24 h later (n = 5 mice for each murine strain). (B) Bacterial load in kidneys. C57BL/6J, A/J, and CSS mice were injected (i.p.) with Sanger 476 (10^7^ CFU/g) and euthanized 24 h later (n = 5 mice for each murine strain). *P*-values were calculated from the F-test. Values of *P*<0.05 were considered significant.

To further understand the basis of the enhanced susceptibility to *S. aureus* infection, we measured the tissue burden of *S. aureus* in A/J and C57BL/6J mice and the CSS mice 24 hours after infection with *S. aureus*. The animals were euthanized and bacterial load in the kidney and peritoneal fluid was determined. The bacterial load in the peritoneal fluid of susceptible A/J mice was significantly higher than in the resistant C57BL/6J mice (178.9±16.3×10^6^ CFU/ml vs. 3.40±1.55×10^6^ CFU/ml; *P* = 0.0001, F-test; Figure 3A). The bacterial load of the peritoneal fluid was also significantly higher in the CSS mice with A/J chromosome 8 (119.3±47.8×10^6^ CFU/ml, *P* = 0.0017, F-test), chromosome 11 (220±74.7×10^6^ CFU/ml, *P* = 0.016, F-test) or chromosome 18 (132.7±25.7×10^6^ CFU/ml, *P* = 0.019, F-test) compared to C57BL/6J mice and to the rest of the chromosome substituted strains (1.531±5.5×10^6^). Similarly, the bacterial load in the kidney of the susceptible A/J mice was significantly higher than in C57BL/6J mice (111.9±99.8×10^6^ CFU/g vs. 33.1±18.7 CFU/g; *P* = 0.003, F-test; Figure 3B). Finally, the bacterial count in the kidney of CSS mice with chromosome 8 (44.8±27.8×10^6^ CFU/g, *P*<0.001, F-test), chromosome 11 (3.5693±0.1940×10^6^ CFU/g, *P*<0.001, F-test) and chromosome 18 (1.3451±0.2491×10^6^ CFU/g, *P* = 0.0013, F-test) was significantly higher than that in C57BL/6J mice and the remainder of the chromosome substituted strains (4.75±86.39×10^3^ CFU/g, *P* = 0.016, F-test). Thus, increased mortality due to *S. aureus* sepsis is associated with a higher bacterial load in the kidneys and peritoneal fluid of susceptible A/J, CSS8, CSS11 and CSS18 mice as compared to the resistant C57BL/6J mice and the remainder of the consomic mouse strains (CSS1–CSS7, CSS9–10, CSS12–CSS17, CSS19, CSSX and CSSY). This correlation of susceptibility with bacterial burden provides important insight into the pathogenesis of early death in murine staphylococcal sepsis, as the genes involved in susceptibility of A/J to *S. aureus* sepsis appear to be associated with a relative inability of the host to control infection.

### Genetic susceptibility to *S. aureus* in mice is not sex linked and has variable forms of inheritance

We next generated F_1_ progeny by crossing the resistant C57BL/6J and susceptible CSS 8, 11, and 18 mice. These crosses result in offspring that, for the relevant chromosome pair (8, 11, or 18), have one C57BL/6J chromosome and one A/J chromosome. Using the same model of infection as described above, we observed no significant difference in the median survival of each F_1_ cohort when the parents were of either sex, indicating that the alleles of the loci mediating susceptibility to *S. aureus* are not imprinted (**Table 2**). F_1_ mice from the C57BL/6J×CSS8 (C8A) and C57BL/6J×CSS18 (C18A) crosses retained susceptibility to *S. aureus* infection (median survival <48 h), whereas F_1_ mice from C57BL/6J×CSS11 cross (C11A) were resistant (median survival >120 h) to *S. aureus* infection. These results indicate that the individual loci mediating susceptibility to *S. aureus* in A/J mice have different modes of inheritance (i.e., autosomal dominant for loci on chromosomes 8 and 18 and autosomal recessive for loci on chromosome 11). Similar to the homozygous CSS mice, the bacterial load in the kidneys of the susceptible F_1_ mice (C8A and C18A) was significantly higher than in the resistant C57BL/6J or C11A mice (data not shown). Taken together, these results provide further evidence that genes governing murine susceptibility to *S. aureus* infection reside on chromosomes 8, 11 and 18.

**Table 2:** Median survivals of F_1_ progeny of Chromosomal Substitution Strains 8, 11, and 18.

F_1_ progeny	Median Survival (d)	Number of Mice Tested (n)
**C57BL/6J (M) X A/J (F)** *	1	10
**A/J (M) X C57BL/6J (F)**	1	10
**C57BL/6J (M) X CSS8 (F)**	2	10
**CSS8 (M) X C57BL/6J (F)**	2	10
**C57BL/6J (M) X CSS18 (F)**	2	10
**CSS18 (M) X C57BL/6J (F)**	2	10
**C57BL/6J (M) X CSS11 (F)**	5	10
**CSS11 (M) X C57BL/6J (F)**	5	10

*Genetic determinants of susceptibility to S. aureus are acquired in a dominant inheritance pattern on chromosomes 8 and 18 and a recessive inheritance pattern on chromosome 11. These data also indicate that the alleles conferring susceptibility to S. aureus in A/J are not imprinted.*

*M = Male; F = Female.

### Genes on chromosomes 8, 11, and 18 are differentially expressed between A/J and C57BL/6J mice

Next, to identify genes on A/J chromosomes 8, 11, and 18 that contribute to susceptibility, we compared whole blood genome transcription profiles of A/J and C57BL/6J mice in both an uninfected state and following *S. aureus* infection. A total of 675 genes on chromosomes 8, 11, and 18 were differentially expressed between uninfected A/J and C57BL/6J mice, and 751, 683, 1223 and 639 were differentially expressed at 2, 4, 6, and 12 hours after infection, respectively. However, only 191 of these genes were differentially expressed at all four post-infection time points (**Table 3** **and Table S1**). Of the 191 genes, 37 genes were similarly expressed in uninfected A/J and C57BL/6J mice. The remaining 155 genes were differentially expressed between C57BL/6J and A/J mice in both uninfected and infected states.

**Table 3:** Number of genes on murine chromosomes 8, 11, and 18 that are differentially expressed between A/J and C57BL/6J at all post-infection timepoints following intraperitoneal infection with *S. aureus*.

Chromosome	Genes on Chromosome	Differentially Expressed Genes at All Post-Infection Time Points*
**8**	1328	56
**11**	2200	100
**18**	683	35
**Total**	4211	191†

*Includes 2 hour, 4 hour, 6 hour, and 12 hour post-infection timepoints.

**†:** *Includes 37 genes (12 on chromosome 8, 20 on chromosome 11, and 5 on chromosome 18) that are differentially expressed between A/J and C57BL/6J only following infection and 154 genes (44 on chromosome 8, 80 on chromosome 11, and 30 on chromosome 18) that are differentially expressed between A/J and C57BL/6J in both uninfected and post-infection states. See Table S1 for complete list of all 191 genes.*

### QTL for susceptibility to *S. aureus* infection on chromosome 18

**Figure 4:**
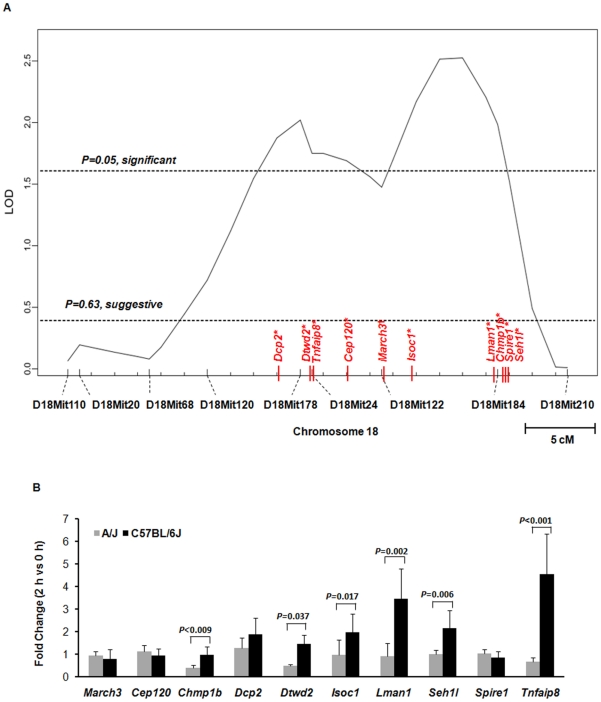
Ten genes on chromosome 18 were identified by both QTL mapping and expression array for susceptibility to *S. aureus*. (A) Chromosome 18 LOD score plot for susceptibility to *S. aureus* in N_2_ backcross mice. Six to eight-week-old backcross mice were injected i.p. with 1×10^7^ CFU/g *S. aureus* and observed every 8 h for 5 days. The horizontal dashed lines indicate the thresholds for significant (*P* = 0.05) and suggestive (*P* = 0.63) LOD score determined by the J/qtl permutation test using 1,000 permuted data sets. The microsatellite markers used for determining genotypes of N_2_ backcross mice are presented along the X-axis. The differentially expressed genes are indicated on chromosome 18. Genes identified within significant or suggestive QTL were indicated with * or /†, respectively. (B) Quantitative real-time PCR validation of 10 genes (*March3*, *Cep120*, *Chmp1b*, *Dcp2*, *Dtwd2*, *Isoc1*, *Lman1*, *Spire1*, *Tnfaip8*, and *Seh1l*) mapping within the two identified QTLs on chr.18 that were also identified by the expression-array selection strategy. Expression values of genes were normalized to 18S rRNA. Six genes (*Dtwd2*, *Tnfaip8*, *Isoc1*, *Lman1*, *Chmp1b*, and *Seh1l*) were significantly differentially expressed between A/J and C57BL/6J infected with *S. aureus* in the significant QTLs on chromosome 18. Values of *P*<0.05 were considered significant (n = 5, paired two-tailed t-test).

To further fine-map the locus (or loci) on chromosome 18 involved in determining susceptibility to *S. aureus* infection, we performed a QTL analysis, generating N_2_ backcross mice by mating F_1_ mice (C18A) to C57BL/6J mice (Figure S1). A total of 144 N_2_ backcross mice were generated and infected with *S. aureus*, and their survival times were measured. No sex-linked susceptibility in N_2_ backcross mice was identified (Figure S2). Using J/qtl software, two significant QTLs were identified as linked to survival time after *S. aureus* infection (Figure 4A), one located at 41 to 54 Mb, and one located at 57 to 67Mb. Approximately 317 genes are contained within these two significant intervals.

Next, we identified genes implicated by both our expression array-based and QTL-based selection strategies. A total of 10 genes (*March3*, *Cep120*, *Chmp1b*, *Dcp2*, *Dtwd2*, *Isoc1*, *Lman1*, *Spire1*, *Tnfaip8*, and *Seh1l*) mapping within our two significant QTLs were differentially expressed between *S. aureus* infected A/J and C57BL/6J mice. Four genes (*Dcp2*, *Dtwd2*, *Tnfaip8*, and *Cep120*) were located within the first significant QTL (41 to 54 Mb) and one gene (*March3*) was located in the suggestive interval for this QTL (56 Mb). The remaining 5 genes (*Isoc1*, *Lman1*, *Chmp1b*, *Spire1*, and *Seh1l*) fell within the second significant QTL (57 to 67 Mb). For 6 of these genes (*Dtwd2*, *Tnfaip8*, *Isoc1*, *Lman1*, *Chmp1b*, and *Seh1l*), real-time PCR confirmed significant differences in expression between *S. aureus*-infected A/J and C57BL/6J mice (*P*<0.05, paired two-tailed t-test) (Figure 4B).

### Assessing biological relevance: Neutrophil and macrophage function in A/J and C57BL/6J

Because of the central role of neutrophils in the host response to *S. aureus*, we considered whether neutrophil dysfunction might be a primary cause of A/J susceptibility to *S. aureus*. To do this, we assessed ex vivo the bactericidal activity of neutrophils from A/J, C57BL/6J, or CSS18 mice by incubating them with *S. aureus*, and then comparing the number of viable bacteria in the extracellular (unphagocytosed) and intracellular (phagocytosed) fractions of neutrophils between mouse strains. As shown in Figure S3A, the percentage of surviving *S. aureus* was similar in both fractions across all three mouse strains. Thus, no significant strain-specific differences in the function or capacity of neutrophils to kill *S. aureus* were noted. We also found no significant strain-specific differences in bactericidal capacity when we repeated this experiment using peritoneal macrophages from A/J and C57BL/6J mice (Figure S3B). Based on these findings, we considered whether the observed susceptibility of A/J mice to *S. aureus* might be due in part to perturbations in the initial host inflammatory response.

### Assessing biological relevance: Cytokine response in A/J and C57BL/6J

**Figure 5:**
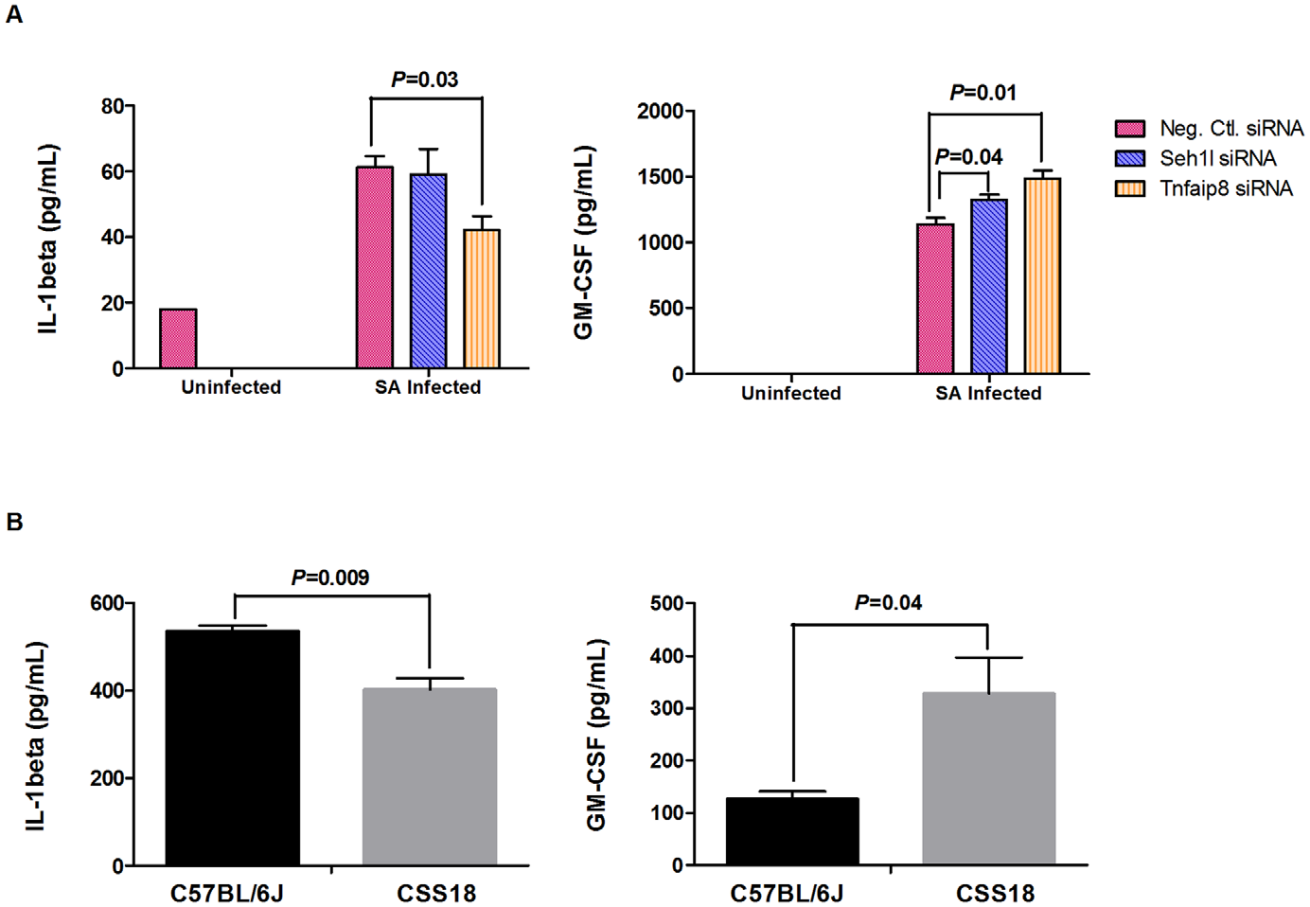
RAW264.7 cells transfected with *Tnfaip8* and *Seh1l* siRNA and peritoneal macrophages from C57BL/6J and CSS18 exhibited significant and consistent patterns of change in the cytokine production (IL-1β and GM-CSF). (A) RAW264.7 cells were treated with negative control, *Tnfaip8* or *Seh1l* siRNA and incubated with *S. aureus* for 5 h. (B) Peritoneal macrophages from CSS18 and C57BL/6J were incubated with *S. aureus* for 48 h. Macrophage culture supernatants were collected at two time points (pre- and post- *S. aureus* stimulation) for cytokine/chemokine analysis. IL-1β and GM-CSF were determined by Luminex-based multiplex cytokine assay. Means and standard deviations of three independent experiments are shown. Values of *P*<0.05 were considered significant (unpaired two-tailed t-test).

To further evaluate the potential importance of our 10 candidate genes in host immune responses to *S. aureus*, we treated mouse macrophage RAW264.7 cells with short interfering RNA (siRNA) oligonucleotides. Knockdown expression of each of the 10 target genes in cells was confirmed by real-time-PCR (Figure S4). The cells were then stimulated with *S. aureus*, and a Luminex-based multiplex cytokine assay was used to evaluate the impact of each genes' individual knockdown expression on the host inflammatory response to *S. aureus*. Cell-culture supernatants from pre- and post- *S. aureus* stimulation conditions were compared (Figure S5). Only TNFAIP8 and SEH1L knockdown cells significantly influenced cytokine levels in *S. aureus*-challenged siRNA-transfected RAW264.7 cells (Figure 5A). Compared to *S. aureus*-challenged negative controls, *S. aureus*-challenged cells with knockdown expression of *Tnfaip8* produced a significantly lower level of IL-1β, while those with knockdown expression of *Seh1l* produced a significantly higher level of GM-CSF.

To evaluate whether our cytokine results from the RAW264.7 knockdown experiments mimicked what was encountered *in vivo*, we first demonstrated by real time PCR that expression levels of *Tnfaip8* and *Seh1l* are significantly lower in whole blood from A/J mice than from C57BL/6J mice (Figure 4B). Next, we confirmed that the same reduced expression pattern was also encountered in peritoneal macrophages (PMΦ) from A/J mice (54±13% [*Tnfaip8*] and 70±10% [*Seh1l*] of C57BL/6J PMΦ mRNA expression, respectively) and CSS18 mice (42±3% [*Tnfaip8*] and 78±5% [*Seh1l*] of C57BL/6J PMΦ mRNA expression, respectively). We then compared cytokine production of PMΦ from C57BL/6J and CSS18 after stimulation with *S. aureus* (Figure S6). We used PMΦ from CSS18 (which contain Chr. 18 from A/J but are otherwise C57BL/6J) in order to eliminate the potential confounding effect on the results of additional unidentified susceptibility genes on Chr. 8 and 11 of A/J mice. As shown in Figure 5B, we encountered the same pattern of IL-1β and GM-CSF in *S. aureus*-stimulated PMΦs from CSS18 mice as we did in the TNFAIP8 and SEH1L knockdown in RAW264.7 cells. Specifically, IL-1β was significantly decreased and GM-CSF was significantly increased in PMΦ of CSS18 compared to that of C57BL/6J (*P* = 0.009 for IL-1β, *P* = 0.04 for GM-CSF; unpaired two tailed t-test).

### Presence of SNPs in the QTL regions

All but one (*Dtwd2*) of the 10 differentially expressed genes in the two QTLs on chr. 18 contained SNPs in the region flanking the first and last exon. Such SNPS could potentially lie within transcriptional regulatory sites. A total of 22 genes within the two QTLs on chromosome 18 contained non-synonymous coding SNPs. These genes included *Dcp2*, *Gm5505*, *Ccdc112*, *Pggt1b*, *Commd10*, *Gm5095*, *Gm3790*, *Ftmt*, *Zfp474*, *Fbn2*, *Slc27a6*, *Gm3957*, *Synpo*, *Tcof1*, *Csf1r*, *Ppargc1b*, *Il17b*, *Afap1l1*, *Sh3tc2*, *Wdr7*, *Alpk2*, *Malt1*, and *5330437l02Rik*.

## Discussion

Host genetic determinants of susceptibility to *S. aureus* are poorly understood. In the current study, we localized the genetic determinants of *S. aureus* susceptibility in A/J inbred mice to chromosomes 8, 11, and 18. From the ∼4200 genes on these three chromosomes, we identified 191 which are differentially expressed between the susceptible A/J and the resistant C57BL/6J inbred mouse strains. We also pursued QTL analysis for chr. 18 to corroborate the findings of our array-based approach with a second strategy. We found two QTLs on chr. 18 over the significance threshold. This suggests that there are at least two genes, one (or more) of which are associated with each peak and which are involved in susceptibility to *S. aureus* infection in A/J mice. In the current report, we have identified two genes, one on each of the two QTL peaks, which are strongly associated with susceptibility to *S. aureus* infection.

We hypothesized that our candidate genes influenced susceptibility to *S. aureus* in A/J mice by regulating macrophage cytokine production. There are several lines of evidence in support of this hypothesis. Macrophages play an important role in initiating host defense to *S. aureus* by secreting cytokines and chemokines [24]–[26], and impairment of this cytokine production by macrophages results in impaired host inflammatory responses [27], [28]. In support of our hypothesis, we found that macrophages from the two mouse lineages did differ significantly in their cytokine response to *S. aureus*. In addition, we found no difference in the bactericidal capacity of either macrophages or neutrophils from A/J and C57BL/6J mice, suggesting that functional deficits within these cells are not the primary cause of the observed differences in susceptibility between the two murine strains. For these reasons, we pursued siRNA-mediated knockdown of our candidate genes in mouse macrophage cells and analyzed the cytokine profiles of knockdown cells exposed to *S. aureus*. As evidenced by real-time PCR, six genes were significantly differentially expressed. More notably, knockdown expression of two of the six genes, *Tnfaip8* and *Seh1l*, significantly altered the specific patterns of cytokine production in *S. aureus*- stimulated RAW cells in a manner that was also observed in PMФ of CSS18, but not C57BL/6J mice. These two genes are prime candidates for influencing susceptibility to *S. aureus*.

Of the 191 genes on chromosomes 8, 11, or 18 that are also differentially expressed between A/J and C57BL/6J mice, 28 have previously been shown to play key roles in the response to various infectious diseases. For example, *Cd209d* has been known to be able to capture Gram-negative bacteria such as *Escherichia coli* and *Salmonella typhimurium* as well as Gram-positive bacteria such as *Streptococcus pneumoniae* [29], [30]. *Tbkbp1* may play a role in antiviral innate immunity as part of the TNF/NFkB interaction pathway [31]. *Map2k3* is induced by various stimuli, including cellular stress, inflammatory cytokines and cell surface receptors, and mediates the activation of p38 mitogen-activated protein kinase (*p38MAPK*) [32]. *Grb2* [33], *Prdx2* [34], and *Ppp2cb* [35] are also involved in the MAPKKK pathway. Activation of the MAPKKK cascade confers resistance to both Gram- negative and Gram-positive pathogens, indicating that signaling pathways initiated by various pathogens converge into a MAPKKK cascade[36], [37]. Interestingly, almost one-third of these known 28 genes were associated with apoptosis (*Birc5*, *Cln8*, *Cyfip2*, *Psme3*, *Rffl*, *Rtn4*, *Trp53*, *Tnfaip8*, and *Xaf1*). Although the precise reasons for the high frequency of apoptosis-related genes are not known, it suggests that this pathway may represent a particularly important area for further study. Finally, our strategy also identified a number of novel genes about which little is known. For example, 20 of the 191 differentially expressed genes on chromosomes 8, 11, and 18 were identified entirely by RIKEN cDNA information, and had no known biological function (**Table S1**).

The TNFAIP8 family of proteins consists of TNFAIP8, TIPE1 (TNFAIP8Like1), TIPE2 and TIPE3. TIPE2 is known as a negative regulator of innate and adaptive immune processes[38]. Woodward et al. showed that TNFAIP8 regulates glucocorticoid-mediated apoptosis of thymocytes [39]. Interestingly, RAW264.7 cells in which TNFAIP8 had been knocked down produced significantly more GM-CSF and significantly less IL-1β compared to control cells. GM-CSF is a critical Th1 cell-derived cytokine that mediates pulmonary inflammation in vivo, and controls neutrophil and macrophage numbers [40], [41]. Interestingly, overexpression of GM-CSF leads to severe inflammation [41]. IL-1β is a key inflammatory cytokine in orchestrating host defense against *S. aureus*, with mice deficient in this cytokine exhibiting markedly increased susceptibility to *S. aureus* [24], [42]. Taken together, *Tnfaip8* is a strong candidate gene governing susceptibility to *S. aureus* through IL-1β and GM-CSF regulation in mice. *Seh1l*, the other candidate gene, is a part of nuclear pore complex Nup107–160 that was recently found to play a role in embryonic development. Although the infectious diseases-related function of this gene is unknown, its knockdown was also shown to increase GM-CSF production in *S. aureus*-stimulated macrophages. Thus, it may be that SEH1L contributes to susceptibility of A/J to *S. aureus* by increasing, along with TNFAIP8, macrophage production of GM-CSF.

The biological relevance of *Tnfaip8* and *Seh1l* is strengthened by several key observations. First, *Tnfaip8* and *Seh1l* exhibited decreased mRNA expression in both peripheral blood and PMΦ of A/J and CSS18 mice as compared with C57BL/6J mice. Second, of the 10 differentially expressed genes on the significant QTL regions of chromosome 18, only *Tnfaip8* and *Seh1l* induced significant changes in cytokine production from siRNA-inhibited, *S. aureus*-challenged macrophages. Third, the cytokines altered by this siRNA-mediated knockdown of TNFAIP8 and SEH1L are critically important components of host response to *S. aureus* [24], [42]. Finally, the cytokine patterns elicited by TNFAIP8 and SEH1L knockdowns were also seen in the peritoneal macrophages of CSS18 mice, but not C57BL/6J mice. Taken together, these data support the importance of our two candidate genes, *Tnfaip8* and *Seh1l*, to the susceptibility to *S. aureus*, both in vivo and in vitro.

Although regulatory variation can play an important role in various complex traits [43]–[46], its role in the current study is unresolved. Nine of the 10 differentially expressed candidate genes on our two QTLs contained SNPs in the region flanking the first and last exon. In addition, 22 other genes on our two QTLs contained non-synonymous coding SNPs. Such SNPs could also potentially contribute to strain-specific phenotypes via modification of protein function. Only 10 of these genes (*Ftmt*, *Fbn2*, *Synpo*, *Tcof1*, *Csf1r*, *Ppargc1b*, *Il17b*, *Sh3tc2*, *Wdr7*, and *Malt1*) were identified in the map of disease genes described in Online Mendelian Inheritance in Man (OMIM). Of these, three (*Csf1r*, *Il17b*, and *Malt1*) are known to be involved in immune response. For example, *Csf1r* encodes a tyrosine kinase growth factor receptor for colony-stimulating factor-1, a cytokine which controls the production, differentiation, and function of macrophages[47]. IL-17b plays an important role in the pathogenesis of inflammatory arthritis [48] in the animal model. MALT1 is essential for NF-kappa-B activation, cytokine production, and proliferation of primary T and B lymphocytes [49]. Future studies will investigate the role of these genes, if any, on the susceptibility of A/J mice to *S. aureus* infection.

The different susceptibilities of A/J and C57BL/6J mice to *S. aureus* infection were recently confirmed by other investigators [3]. von Kockritz-Blickwede et al. suggested that resistance to *S. aureus* in C57BL/6 mice is critically dependent on an effective and fast recruitment of neutrophils to the site of infection due to different kinetic profiles in expression of KC (*Cxcl1*) and MIP-2 (*Cxcl2*). These two chemokines play a central role in mediating neutrophil recruitment to sites of infection by binding to receptors on the surface of these cells. In support of this hypothesis, we found that *Cxcl1* was significantly differentially expressed between A/J and C57BL/6J mice at 2, 6, and 12 hours after infection with *S. aureus*. Although the gene coding for *Cxcl1* is located on mouse chromosome 5, CSS5 mice infected with *S. aureus* in the current study retained the resistant phenotype. This finding suggests that genes on mouse chromosome 5 are not directly responsible for A/J's susceptibility, but that the reduced chemokine production observed in *S. aureus*- infected A/J mice [3] may be regulated by one or more genes on chromosomes 8, 11, or 18.

Although C5 deficiency has been shown to contribute to susceptibility to a variety pathogens, including *Pseudomonas aeruginosa*, *Mycobacterium tuberculosis*, *Listeria monocytogenes*, and *Candida albicans* [22], [50]–[53], its contribution to susceptibility to *S. aureus* is unresolved. For example, Crequetti et al. (1983) showed that C5-deficient mice had impaired lung clearance of *S. aureus* [53], while Toews and Pierce (1984) demonstrated similar lung clearance of *S. aureus* and PMN recruitment in both C5-deficient and sufficient mice [54]. In addition, Easmon and Glynn reported decreased survival of C5-deficient mice compared with C5-sufficient controls after intraperitoneal injection with mucoid *S. aureus* strains [55], while Cunnion et al. observed similar survival in both C5-deficient and sufficient mice challenged via intravenous injection with *S. aureus* [56]. Finally, von Kockritz-Blickwede and colleagues recently demonstrated a protective role of complement C5a in an experimental model of *S. aureus* bacteremia [23]. In the present study, all three of the additional C5-deficient mouse strains we tested were resistant to *S. aureus*. Our consomic experiments also suggest that C5-deficiency is not the primary determinant of susceptibility to *S. aureus* in A/J mice. Although the gene for C5 (Hc) resides on chromosome 2, CSS2 mice (which contain A/J chromosome 2 and thus would be C5 deficient) remained resistant to *S. aureus* infection. Based on this body of evidence, we conclude that factors other than C5-deficiency are likely to be primarily responsible for the susceptibility to *S. aureus* seen in A/J mice.

The current study has limitations. First, linkage scores of the two QTLs on chr. 18 imply that there may be more than one gene on the larger significant peak that plays a role in susceptibility to *S. aureus*. One possible additional susceptibility gene is *Lman1*. In the Figure S5, knockdown of LMAN1 showed dramatically reduced IL-1α compared to control when stimulated with *S. aureus*. Although it was not statistically significant (*P* = 0.08), the quantity of IL-1α in LMAN1 knockdown cells was below the detection value of standard minimum. Thus, it is possible that testing of a much larger number of samples would reveal a statistical significance of additional candidate genes such as *Lman1* in the larger peak. Second, there may be genes on other chromosomes that contribute to a lesser extent, or that have a strong effect only when acting in conjunction with genes on another chromosome. Third, our strategy will miss genes on chromosomes 8, 11, or 18 that contribute to phenotype at the protein level (e.g., coding SNPs) or by modulating the gene expression patterns of genes on other chromosomes. Fourth, our strategy would not allow detection of polymorphic regulatory sites more than 2kb away from genes, such as those within gene deserts. Finally, we have only completed QTL linkage analysis for chr. 18. Thus, additional work is required, including QTL mapping analysis for A/J chromosomes 8 and 11, defining the pathogenesis of *Tnfaip8* and *Seh1l* using knockout mice, and evaluating the impact of *Tnfaip8* and *Seh1l* on host susceptibility to different pathogen classes. Ultimately, the importance of the identified candidate genes, or their human homologs, will need to be evaluated in patients with *S. aureus* infections.

Despite these limitations, this study makes several key observations. First, using expression array-based strategies, we have identified 191 genes that are differentially expressed between susceptible and resistant mice, and are found on three different chromosomes where loci conferring susceptibility to *S. aureus* infection have been mapped. Second, we have identified two QTLs on chr. 18 that are linked to survival time after infection with *S. aureus*. Ten differentially expressed genes mapped to the significant- or suggestive threshold of these two QTLs. Of these 10 genes, two (*Tnfaip8* and *Seh1l*) significantly affect the production of IL-1β and GM-CSF in *S. aureus*-stimulated mouse macrophages in a pattern also seen in peritoneal macrophages from mice containing A/J chromosome 18, thereby supporting a potential role for these two genes in host response to *S. aureus*. These genes in general, and *Tnfaip8* and *Seh1l* in particular, represent promising candidates for the genetic basis of host susceptibility to this serious, common pathogen.

## Materials and Methods

### Ethics statement

All animal experiments were performed in accordance to NIH guidelines, the Animal Welfare Act, and US federal law. Such experiments were carried out following approval by the Duke University's Institutional Animal Care & Use Committee (IACUC) which has been accredited by the Association for Assessment and Accreditation of Laboratory Animal Care (AAALAC) International. All animals were housed in a centralized and AAALAC-accredited research animal facility that is fully staffed with trained husbandry, technical, and veterinary personnel.

### Mouse strains

C57BL/6J and A/J mice were purchased from The Jackson Laboratory (Bar Harbor, ME). Chromosome substitution strains (CSS) were generated by transferring a single, full-length chromosome from the donor strain (A/J) onto the genetic background of the host strain (C57BL/6J) by repeated backcrossing [15]. Male CSS mice (CSS1–19, CSSX and CSSY) were also obtained from the Jackson Laboratory (Bar Harbor, ME).

### Preparation of bacterial cells

*S. aureus* clinical strains, Sanger 476 or MW2 were used in the mortality studies. For preparation of *S. aureus* for injection, an overnight bacterial culture of *S. aureus* was diluted with fresh tryptic soy broth (TSB) and incubated (37°C) with aeration to log-phase growth (optical density at wavelength 600 nm (O.D.600) of ∼1.0) [57]. *S. aureus* was harvested by centrifugation, rinsed, and resuspended in saline. To mimic the natural course of *S. aureus* infection in humans, which typically arises from a primary focus of infection and disseminates to other sites, we employed an intraperitoneal (i.p.) route of infection in our animal model [58].

### Murine phenotype definitions, injection (*S. aureus*), and specimen procurement

Mouse phenotypes were defined as either ***resistant*** (survival for >120 hours following *S. aureus* infection) or ***susceptible*** (survival for <48 hours following *S. aureus* infection). Mice were injected via an intraperitoneal (i.p.) route with 1×10^7^ CFU/g *S. aureus* depending on the experiment and observed every 8 h for morbidity. Mice were sacrificed using CO_2_ asphyxiation if they appeared moribund. Pain/distress was assessed using a numerical scale for the following characteristics: Appearance (0 = normal; 1 = lack of grooming; 2 = rough hair coat; 3 = abnormal posture); Behavior (0 = normal; 1 = moving slowly; 2 = moving slowly, irregular ambulation; 3 = immobile). A score of 3 indicated significant pain and distress and culminated in the early euthanasia of the animal. Peritoneal lavage was performed as described previously [2]. Kidneys were harvested and either frozen (−80°C) or fixed in 10% buffered formalin, as appropriate, for subsequent analysis. For experiments involving RNA analysis, blood was collected by intracardiac puncture and stored in RNAlater at −20°C.

### Quantifying bacterial load in murine tissues and blood

Kidneys collected from euthanized animals were homogenized in phosphate buffered saline (PBS) and diluted 10 fold serially. Peritoneal lavage (50 µl) was serially diluted in PBS. The serial dilutions (50 µl) were plated in Tryptic Soy Agar (TSA) plates and incubated (37°C, overnight) to count the number of colony forming units (CFU) of *S. aureus*.

### Generation of F_1_ and N_2_ progeny

For the current study, susceptible CSS mice with chromosome 8, 11 and 18 from A/J mice on a C57BL/6J background (CSS8, CSS11 and CSS18) were mated with resistant C57BL/6J strain in reciprocal crosses [C57BL/6J male×CSS8, 11 or 18 female) and (CSS8, 11 or 18 male×C57BL/6J female)] to generate an F_1_ population that was heterozygous for the chromosome of interest on an otherwise uniform C57BL/6J genomic background. To generate N_2_ backcross mice for QTL linkage analysis on chromosome 18, we bred F_1_ mice (C18A) to C57BL/6J (Figure S1).

### Microarray

Total RNA was prepared from mouse blood using the Mouse RiboPure Blood RNA isolation kit (Ambion) following the manufacturer's instruction. Globin mRNA was removed from whole blood RNA samples using the Globinclear kit (Ambion). A total of 30 samples passed the quality criteria of the Agilent Bioanalyzer and were used for microarray analysis. Since the total RNA yield of many samples was low, 1 round of linear amplification was performed for all samples (Ambion MessageAmp Primier). Mouse Genome 430 2.0 Array-Chips were used (Affymetrix). The biotin-labeled cDNA was hybridized to the arrays for 16 hours at 45°C following the manufacturer's instruction. The arrays were then washed and labeled with streptavidinphycoerythrin (strep-PE), and the signal was amplified using biotinylated antistreptavidin followed by another round of staining with strep-PE. Washing and staining were performed on the Affymetrix fluidics station according to the recommended fluidics protocol. Labeled gene chips were scanned using an Affymetrix Genechip Scanner 7G (Affymetrix). This array contains 45,101 probe sets to analyze the expression level of over 39,000 transcripts and variants from over 34,000 well characterized mouse genes. The microarray data have been deposited in the NCBI GEO and are accessible through GEO series accession no. GSE19668.

### Analysis of gene expression microarray data

To identify genes for which differential expression between A/J and C57BL/6J mice could contribute to host susceptibility to *S. aureus* infection, we compared the gene expression profiles between uninfected A/J and C57BL/6J mice and between infected A/J and C57BL/6J mice at 2, 4, 6, and 12 hours after infection. We used a multi-step approach that included i) preprocessing to determine probeset expression values, ii) filtering based on probeset annotations, iii) identification of genes with statistically significant differential expression between A/J and C57BL/6J mice after infection, and iv) ranking based on fold change (FC) in gene expression and Gene Ontology (GO) (http://www.geneontology.org) annotation.

Preprocessing was conducted using the Robust Multichip Analysis (RMA) [59] implementation in the Bioconductor “affy” package (http://www.bioconductor.org/), with an additional step to account for differences in probe hybridization resulting from single nucleotide polymorphisms (SNP) between A/J and C57BL/6J mice. The additional step is referred to as SNP masking [60] and is applied after background correction and quantile-quantile normalization but prior to the determination of probeset expression values. Genomic locations hybridized by each probe were obtained from the Ensembl database (http://www.ensembl.org/index.html), and these genomic locations were compared to the locations of SNPs for which A/J and C57BL/6J mice have different alleles. Probes that hybridize to such SNPs within their target transcripts were excluded from the determination of probeset expression values. There were 7714 probesets for which some of the probes were excluded. In addition, there were four probesets excluded from further analysis because all probes in the set hybridize to SNPs: 1437478_s_at, 1416030_a_at, 1452066_a_at, and 1449635_at. SNP data were obtained from the Perlegen SNP database (http://mouse.perlegen.com/mouse/index.html).

We next filtered the probesets based on probeset annotations. We eliminated from further analysis 6041 probesets with no gene symbol annotation, 2441 probesets with “_x_at” in the probeset name, and 2091 probesets with “_s_at” in the probset name (including 1437478_s_at mentioned above). Probesets with the “_x_at” designation are those that are known to cross-hybridize in an unpredictable manner. Probesets with the “_s_at” designation are those for which multiple different probesets have probes in common.

The remaining 34,706 probesets were analyzed using Analysis of Variance (ANOVA) to determine whether the difference in mean expression levels between A/J and C57BL/6J mice are statistically significant. The following generalized linear model was used:
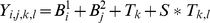
where B^1^ corresponds to IVT batch effects, B^2^ to hybridization batch effects, T to time main effects, and S*T to strain-time interaction effects, where the time factor levels correspond to the uninfected state and 2, 4, 6, and 12 hours post-infection). A list of candidate genes for further analysis was compiled by identifying those genes on chromosomes 8, 11, or 18 with p<0.05 for the S*T model coefficient for all post-infection times (i.e. 2, 4, 6, and 12 hours post-infection). There are 191 such genes (**Table S1**). We have not used a generalized family-wise error rate (gFWER), as stringent *P*-value thresholds have been shown to reduce the reproducibility of lists of differentially expressed genes [61]. We have chosen, therefore, to use non-adjusted p values and to further refine the candidate gene list based on biological rather than statistical criteria, namely differential expression between strains for multiple post-infection timepoints, as well as GO annotations and FC as discussed below. The estimated false discovery rate ranges from 0.09 (6 hours post-infection) to 0.3 (12 hours post-infection) [62].

### SNPs allele differences between A/J and C57BL/6J

Single nucleotide polymorphism (SNP) data were obtained from the Mouse Genome Informatics database (MGI) (http://www.informatics.jax.org/) for the A/J and C57BL/6J mouse strains [63] for all genes on the candidate gene list. We identified those SNP loci within the gene boundaries or within 2 kilobases of the gene boundaries and which have allele differences between the two strains. We next identified the functional class of each SNP as assigned in the MGI database. MGI assigns functional classes according to those defined by dbSNP (http://www.ncbi.nlm.nih.gov/projects/SNP/). The functional classes are: coding-nonsynonymous, coding-synonymous, intron, locus-region, splice-site, mRNA-UTR, contig-reference, coding, and coding-exception. SNPs in untranscribed regions but within 2 kilobases of the 5′ boundary of a gene or within 0.5 kilobases of the 3′ boundary of a gene are assigned the locus-region functional class. SNPs that lie within promoter or enhancer sites are therefore most likely assigned the locus-region functional class.

### QTL linkage analysis

Polymorphic microsatellite markers for the cross between C57BL/6J and A/J were chosen from a database maintained by Mouse Genomic Informatics (http://www.informatics.jax.org/). To cover chromosome 18, nine microsatellite marker were selected with an average inter-marker distance of 5.3 cM. A total of 144 N_2_ backcross were generated, all of which were genotyped for each microsatellite marker by PCR amplification and gel electrophoresis. PCR genotyping reactions were used Platinum Taq polymerase (Invitrogen Co.). J/qtl software was used to analyze phenotype and genotype data for association between survival time after infection with *S. aureus* and marker location. Phenotypes were defined as either sensitive or resistant based on the dichotomization of survival data (Survival of less than 1day is “0” and survival of longer than 1.5 days is “1”, respectively). Because our analyses focused solely upon one chromosome, our significance threshold reflected a chromosome – level analysis [64]–[66], rather than a genome-wide approach [67]. All linkage analysis results were expressed as LOD scores. LOD score was considered “suggestive” if > = 0.41 (*P* = 0.63) and “Significant” if > = 1.64 (*P* = 0.05). Threshold values for linkage were determined by a 1,000 permutation test by using J/qtl.

### Isolation of bone marrow-derived neutrophils

Bone marrow-derived neutrophils were isolated as described previously [2]. Briefly, bones from the femurs and tibias of 8-week-old male A/J and C57BL/6J mice were removed of all muscles. The bone marrow cells were flushed out with Hank's buffer (without calcium and magnesium) and filtered through a 70-µm nylon cell strainer to remove cell clumps. Neutrophils were separated from the remaining cells by centrifugation over discontinuous Percoll (Amersham Biosciences) gradients at 500×*g* for 30 min at 4°C, consisting of 52% (vol/vol), 69% (vol/vol), and 78% (vol/vol) Percoll in PBS. Neutrophils from the interface of the 65 and 75% fractions were washed with Hank's buffer (without calcium and magnesium) and cultured (37°C, 10% CO_2_) in RPMI 1640 supplemented with 10% fetal calf serum (both from the American Type Culture Collection).

### Neutrophil and macrophage bactericidal assay

Killing of *S. aureus* was assayed as described [68] with modification. *S. aureus* cells were opsonized with 10% mouse serum at 37°C for 30 min and washed twice in PBS. In brief, 1 ml of the RPMI 1640 supplemented with 10% fetal calf serum containing neutrophils (2×10^6^ per ml) and *S. aureus* (2×10^6^/ ml) was placed in the sterile 1.5 ml tubes. The tubes were incubated at 37°C with end-over-end rotation (6 rpm). After 90 min of incubation, the neutrophils were centrifuged at 100×g for 5 min at 4°C and washed twice with 1 ml of cold PBS. Supernatants were used for evaluation of the count of extracellular bacteria. The neutrophil pellet was lysed in sterile water (pH 11) to release intracellular bacteria. The each resulting suspension was serially diluted in PBS and bacterial numbers were determined after plating onto TSB agar. Peritoneal macrophage bactericidal assay was performed in the same manner as neutrophil bactericidal assay except the final step for bacterial counting [69], [70]. After 90 min of incubation, the each sample was diluted in sterile water for 5 min to lyse the macrophage, and serially diluted in PBS. The dilutions were plated on TSB agar, and the plates were incubated overnight at 37oC. The amount of killing was determined by plate count of surviving bacteria (extracellular and intracellular bacteria).

### Small interfering RNA (siRNA) experiments

To test the role of each candidate gene on cytokine production by host defense cells, we transfected siRNAs into the mouse macrophage cell line RAW264.7. All siRNAs were purchased from Ambion. Cells were transfected by using siPORT *Amine* Transfection Agent in 48-well plate (Ambion), according to the manufacturer's instructions. Transfections were carried out using 30 nM siRNA and 400,000 cells per well. Cells were maintained in DMEM supplemented with 10% FBS. Forty-eight hours after transfection, *S. aureus* Bioparticles (Invitrogen) was added to a final concentration of 10 µg/ml. After 5 h, supernatants (medium) and cells were collected, stored at −70°C for multiplex cytokine assay (described below) and real-time PCR analyses, respectively. RNA was isolated for detecting the level of mRNA expression. The target gene mRNA expression was analyzed by real-time PCR (TaqMan) using 1 µg total RNA from untreated cells or from cells treated with oligonucleotides. The reaction was performed using the High-Capacity cDNA Reverse Transcription kit (Applied Biosystems) and was followed by Taqman real-time PCR. A full list of gene names, siRNA ID numbers, sequences, and Taqman assay ID numbers are shown in Table S2.

### Peritoneal macrophage (PMΦ) isolation and stimulation

Resident PMΦs were obtained by lavage of the peritoneal cavity of uninfected mice (CSS18 and C57BL/6J) as described elsewhere [69], [71]. Cells were washed once with PBS by centrifugation (300×g, 5 min at 4°C) and resuspended at a concentration of 1×10^6^ cells/ml of RPMI1640 containing 10% FBS. Cells were incubated in a 24-well plate for 2 h and the remaining nonadherent cells were removed by washing with media. The macrophage monolayers were then stimulated to produce cytokines by incubation with 10 µg of *S. aureus* Bioparticles for 48 h at 37°C. Incubation time was determined based on the previous similar studies [72], [73]. Culture supernatants for Luminex assay were collected pre- and post- *S. aureus* stimulation.

### Measurement of cytokine/chemokine production

Cytokine production was assayed from the collected supernatant of the *S. aureus*-challenged siRNA transfected macrophages and the *S. aureus*-challenged peritoneal macrophages from C57BL/6J and CSS18 using a multiplex cytokine assay kit and Luminex technology (Bio-Rad) available at Duke Human Vaccine Institute. Twenty cytokines were tested: FGF basic, TNFα, IL-1α, IL-1β, IL-2, IL-4, IL-5, IL-6, IL-10, IL-12p40/p70, IL-13, IL-17, IFNγ, IP-10, KC, MIG, MIP-1α, MCP-1, VEGF, and GM-CSF. Of the 20 tested cytokines, 9 (FGF basic, IFNγ, IL-2, IL-4, IL-5, IL-13, IL-17, MIG, and IP-10) were excluded from our analysis due to weak signal in both pre- and post- *S. aureus* stimulated supernatant (5 cytokines); lack of response to *S. aureus* (2 cytokines), and cross-contamination (2 cytokines) (Figure S5). The concentrations for cytokines and chemokines were compared between two groups (negative control vs target gene) with a two-tailed t-test for unpaired samples. Differences were considered to be statistically significant when the *P*-value was smaller than 0.05.

## Supporting Information

Figure S1 Breeding scheme used to generate the chromosome 18 backcross mapping. F1 mice (C18A) generated by crossing CSS18 with C57BL/6J. Recombinant N_2_ backcross mice made by mating F1 (C18A) mice to the C57BL/6J inbred mouse strain. A total 144 N_2_ backcross mice were generated and used genotyped using microsatellite markers and phenotyped for susceptibility to *S. aureus* infection. In the schematic diagram of the genome, chromosomes from C57BL/6J and A/J are indicated by blue- and red-colored boxes, respectively.
(1.04 MB TIF)

Figure S2 No sex-linked survival in N_2_ backcross for chromosome 18 mice was identified. A total of 144 N_2_ backcross mice, generated by crossing C18A with C57BL/6J, were injected i.p. with 1×10^7^ CFU/g *S. aureus* and observed every 8 h.
(0.49 MB TIF)

Figure S3 Bactericidal activities of neutrophils and peritoneal macrophages isolated from A/J and C57BL/6J mice are indistinguishable. (A) Neutrophils (2×10^6^) from bone marrow of A/J, C57BL/6J, or CSS18 mice were incubated with 2×10^6^ CFU of *S. aureus* with end-over-end rotation for 90 min at 37°C. Neutrophils were centrifuged at 100×g for 5 min at 4°C and washed twice with PBS. Supernatants were used for evaluation of the count of extracellular viable bacteria. Neutrophil pellets were disrupted with sterile water (pH 11) to release and quantify intracellular surviving bacteria. Bars represent the mean ± SD of three individual mice. Values of *P*<0.05 were considered significant (unpaired two-tailed t-test). (B) Peritoneal macrophages (2×10^6^) from C57BL/6J and A/J mice were incubated with 2×10^6^ CFU of *S. aureus* with end-over-end rotation for 90 min at 37°C. The each sample was diluted in sterile water to release and quantify surviving bacteria. Bars represent the mean ± SD of three individual mice. Values of *P*<0.05 were considered significant (unpaired two-tailed t-test).
(0.83 MB TIF)

Figure S4 Knockdown efficiencies of the 10 target genes in RAW264.7 cells by real-time PCR. mRNA expression levels of each gene in wild-type (WT) and knockdown (KD) RAW264.7 macrophage were determined by real-time PCR. Real-time PCR measurements are normalized to GAPDH mRNA.
(1.22 MB TIF)

Figure S5 Cytokine/chemokine profiles from the knockdowns of the 10 target genes in RAW264.7 cells pre- and post- *S. aureus* stimulation. RAW264.7 macrophage cells were transfected with siRNA for each candidate gene or negative control siRNA (30nM). At 48 h after transfection, the levels of cytokines/chemokines in *S. aureus*-stimulated cell culture supernatants were measured by Luminex-based multiplex cytokine assay. The horizontal dashed lines indicate the minimum detectable concentration. Means and standard deviations of three independent experiments are shown. Values of *P*<0.05 were considered significant (unpaired two-tailed t-test).
(6.96 MB TIF)

Figure S6 Cytokine/chemokine profiles of peritoneal macrophage from Chromosomal Substitution Strain 18 (CSS18) and C57BL/6J mice pre- and post- *S. aureus* stimulation. CSS18 mice, which contain A/J chromosome 18 but are otherwise genetically C57BL/6J, were used to minimize potential confounding effects of genetic susceptibility factors on A/J chromosomes 8 and 11. The peritoneal macrophage cells were isolated from C57BL/6J and CSS18 and cultured in RPMI1640 containing 10% FBS for 2 h. The remaining nonadherent cells were removed by washing with media and the macrophage monolayers were then stimulated to produce cytokine by incubation with 10 µg of *S. aureus* Bioparticles for 48 h at 37°C. The levels of cytokines/chemokines in cell culture supernatants at both pre- and post- stimulated conditions were measured by Luminex-based multiplex cytokine assay. The horizontal dashed lines indicate the minimum detectable concentration. Means and standard deviations of three independent experiments are shown. Values of *P*<0.05 were considered significant (unpaired two-tailed t-test).
(3.52 MB TIF)

Table S1 Genes differentially expressed between A/J and C57BL/6J mice at 2, 4, 6, and 12 hours after infection with *S. aureus*. The gene symbol for each of the 191 differentially expressed genes is shown, along with its gene name, chromosome location, human homologue, and fold change and *P*-value for differential expression at 2 hours post infection. Fold change is shown as the mean expression level in A/J mice divided by the mean expression level in C57BL/6J mice. For genes with multiple probesets, the fold change and *P*-value are shown for the probeset with largest fold change at 2 hours post infection. All gene information was taken from the Mouse Genome Informatics (MGI) (http://www.informatics.jax.org/) and National Center for Biotechnology Information (NCBI) (http://www.ncbi.nlm.nih.gov/) databases. * Genes do not have SNP differences identified between A/J and C57BL/6J.
(9.73 MB TIF)

Table S2 List of siRNAs and Taqman assay used in this study.
(2.08 MB TIF)
