# Supplementary figures and images for: Two Genes on A/J Chromosome 18 Are Associated with Susceptibility to Staphylococcus aureus Infection by Combined Microarray and QTL Analyses
Source: PLoS Pathog. 2010 Sep 2;6(9):e1001088. doi: 10.1371/journal.ppat.1001088 (PMC2932726; doi:10.1371/journal.ppat.1001088)

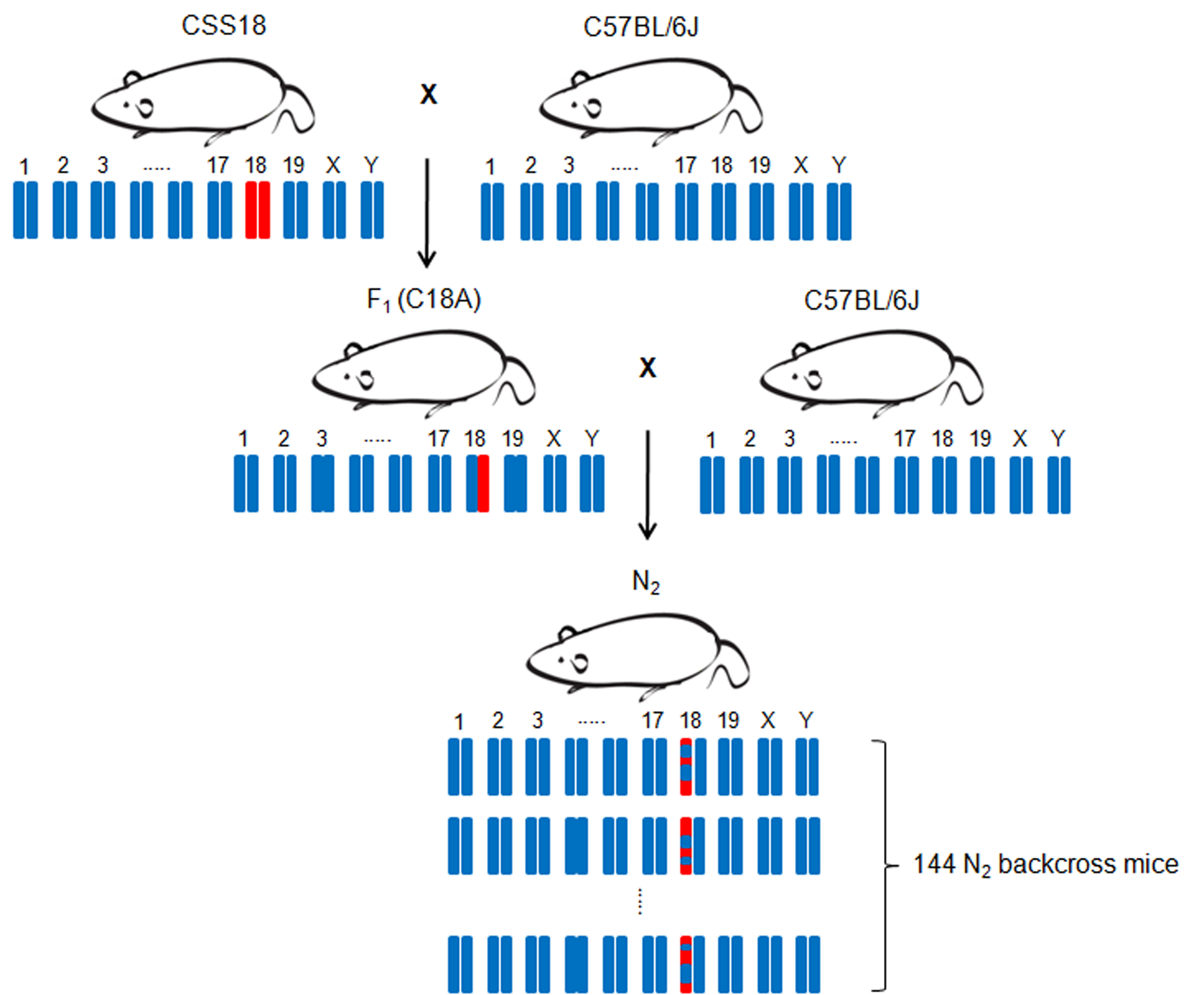

Supplement: Figure S1 — Breeding scheme used to generate the chromosome 18 backcross mapping. F1 mice (C18A) generated by crossing CSS18 with C57BL/6J. Recombinant N2 backcross mice made by mating F1 (C18A) mice to the C57BL/6J inbred mouse strain. A total 144 N2 backcross mice were generated and used genotyped using microsatellite markers and phenotyped for susceptibility to S. aureus infection. In the schematic diagram of the genome, chromosomes from C57BL/6J and A/J are indicated by blue- and red-colored boxes, respectively. (1.04 MB TIF) [file ppat.1001088.s001.tif]

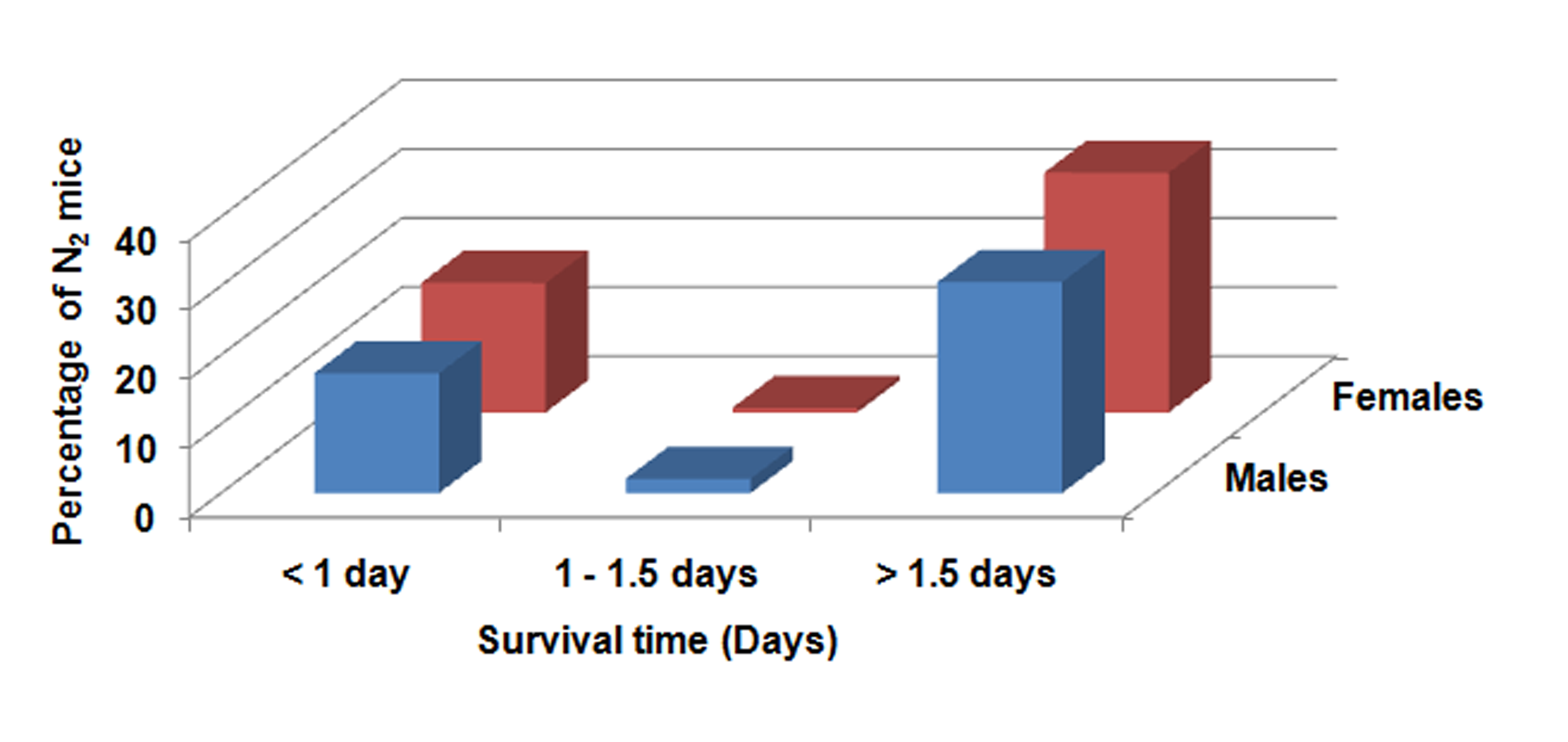

Supplement: Figure S2 — No sex-linked survival in N2 backcross for chromosome 18 mice was identified. A total of 144 N2 backcross mice, generated by crossing C18A with C57BL/6J, were injected i.p. with 1×10^7^ CFU/g S. aureus and observed every 8 h. (0.49 MB TIF) [file ppat.1001088.s002.tif]

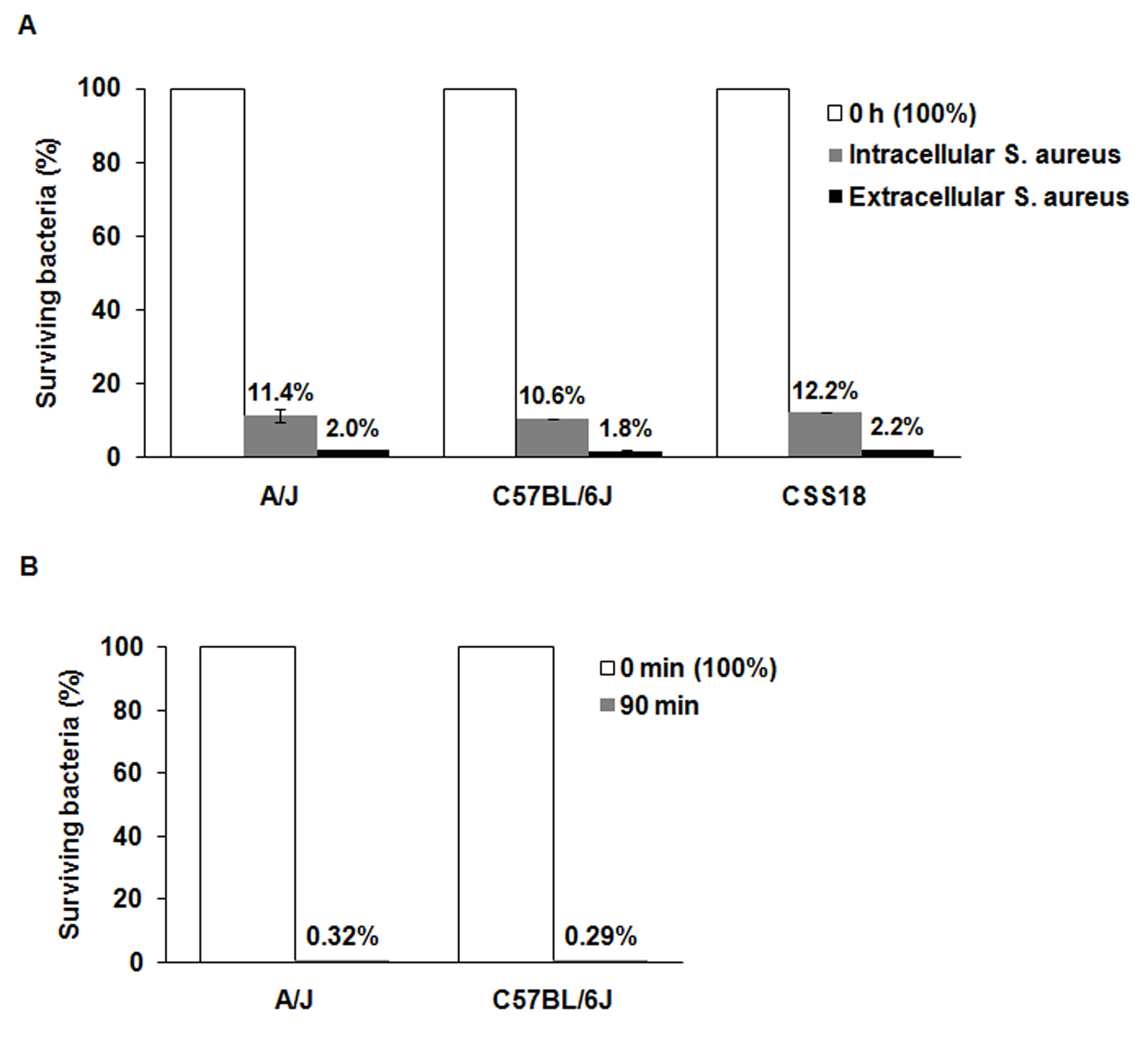

Supplement: Figure S3 — Bactericidal activities of neutrophils and peritoneal macrophages isolated from A/J and C57BL/6J mice are indistinguishable. (A) Neutrophils (2×10^6^) from bone marrow of A/J, C57BL/6J, or CSS18 mice were incubated with 2×10^6^ CFU of S. aureus with end-over-end rotation for 90 min at 37°C. Neutrophils were centrifuged at 100×g for 5 min at 4°C and washed twice with PBS. Supernatants were used for evaluation of the count of extracellular viable bacteria. Neutrophil pellets were disrupted with sterile water (pH 11) to release and quantify intracellular surviving bacteria. Bars represent the mean ± SD of three individual mice. Values of P<0.05 were considered significant (unpaired two-tailed t-test). (B) Peritoneal macrophages (2×10^6^) from C57BL/6J and A/J mice were incubated with 2×10^6^ CFU of S. aureus with end-over-end rotation for 90 min at 37°C. The each sample was diluted in sterile water to release and quantify surviving bacteria. Bars represent the mean ± SD of three individual mice. Values of P<0.05 were considered significant (unpaired two-tailed t-test). (0.83 MB TIF) [file ppat.1001088.s003.tif]

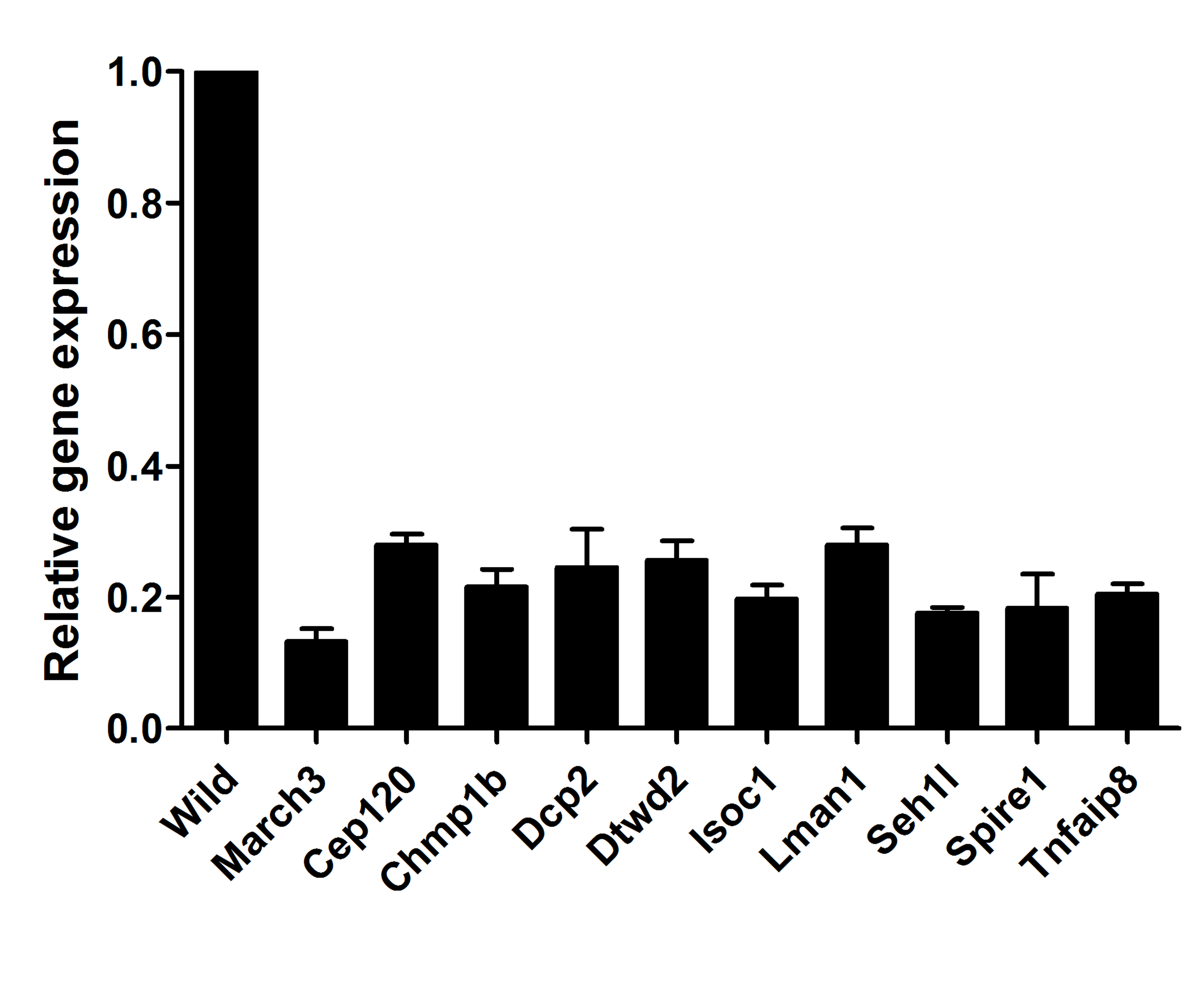

Supplement: Figure S4 — Knockdown efficiencies of the 10 target genes in RAW264.7 cells by real-time PCR. mRNA expression levels of each gene in wild-type (WT) and knockdown (KD) RAW264.7 macrophage were determined by real-time PCR. Real-time PCR measurements are normalized to GAPDH mRNA. (1.22 MB TIF) [file ppat.1001088.s004.tif]

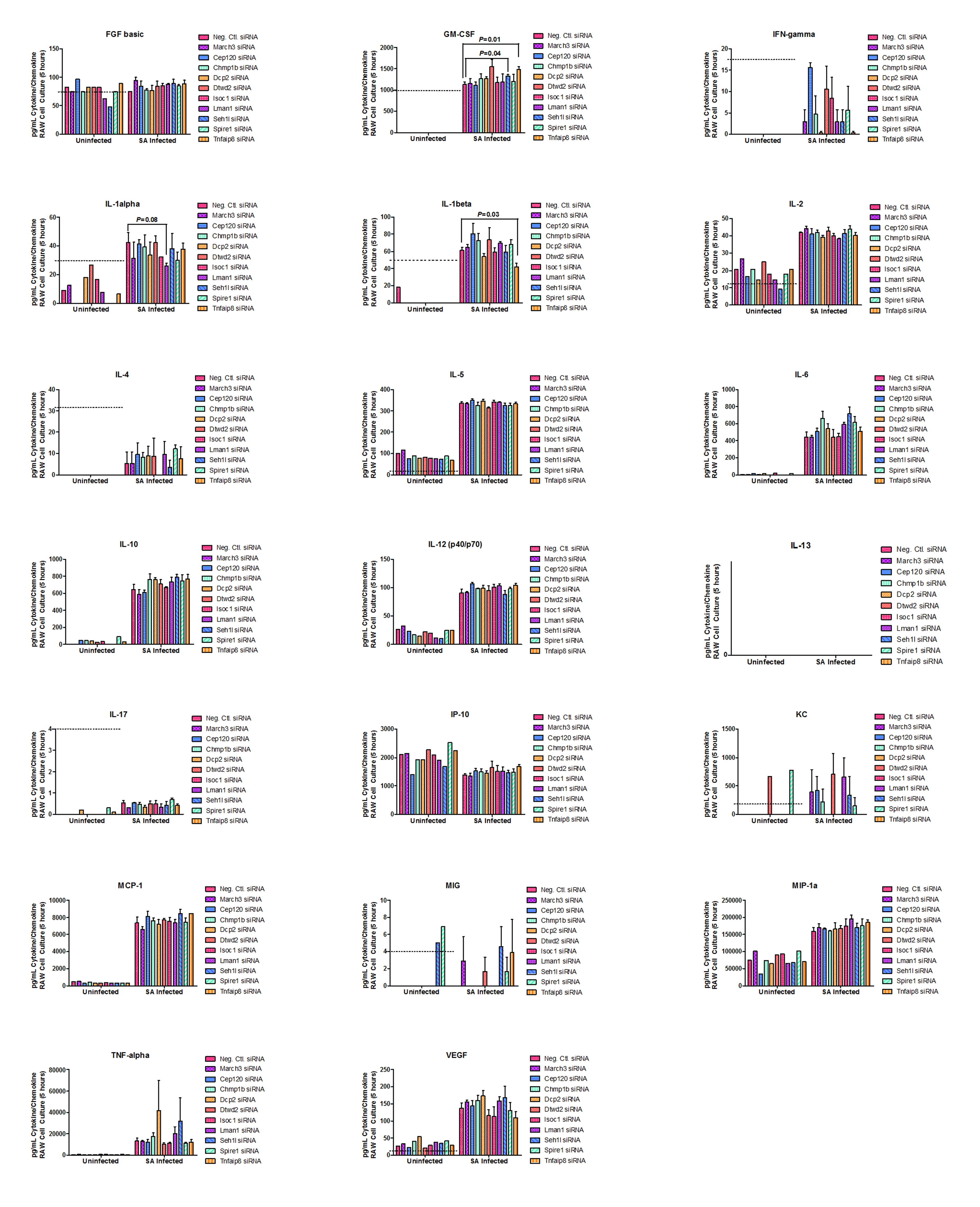

Supplement: Figure S5 — Cytokine/chemokine profiles from the knockdowns of the 10 target genes in RAW264.7 cells pre- and post- S. aureus stimulation. RAW264.7 macrophage cells were transfected with siRNA for each candidate gene or negative control siRNA (30nM). At 48 h after transfection, the levels of cytokines/chemokines in S. aureus-stimulated cell culture supernatants were measured by Luminex-based multiplex cytokine assay. The horizontal dashed lines indicate the minimum detectable concentration. Means and standard deviations of three independent experiments are shown. Values of P<0.05 were considered significant (unpaired two-tailed t-test). (6.96 MB TIF) [file ppat.1001088.s005.tif]

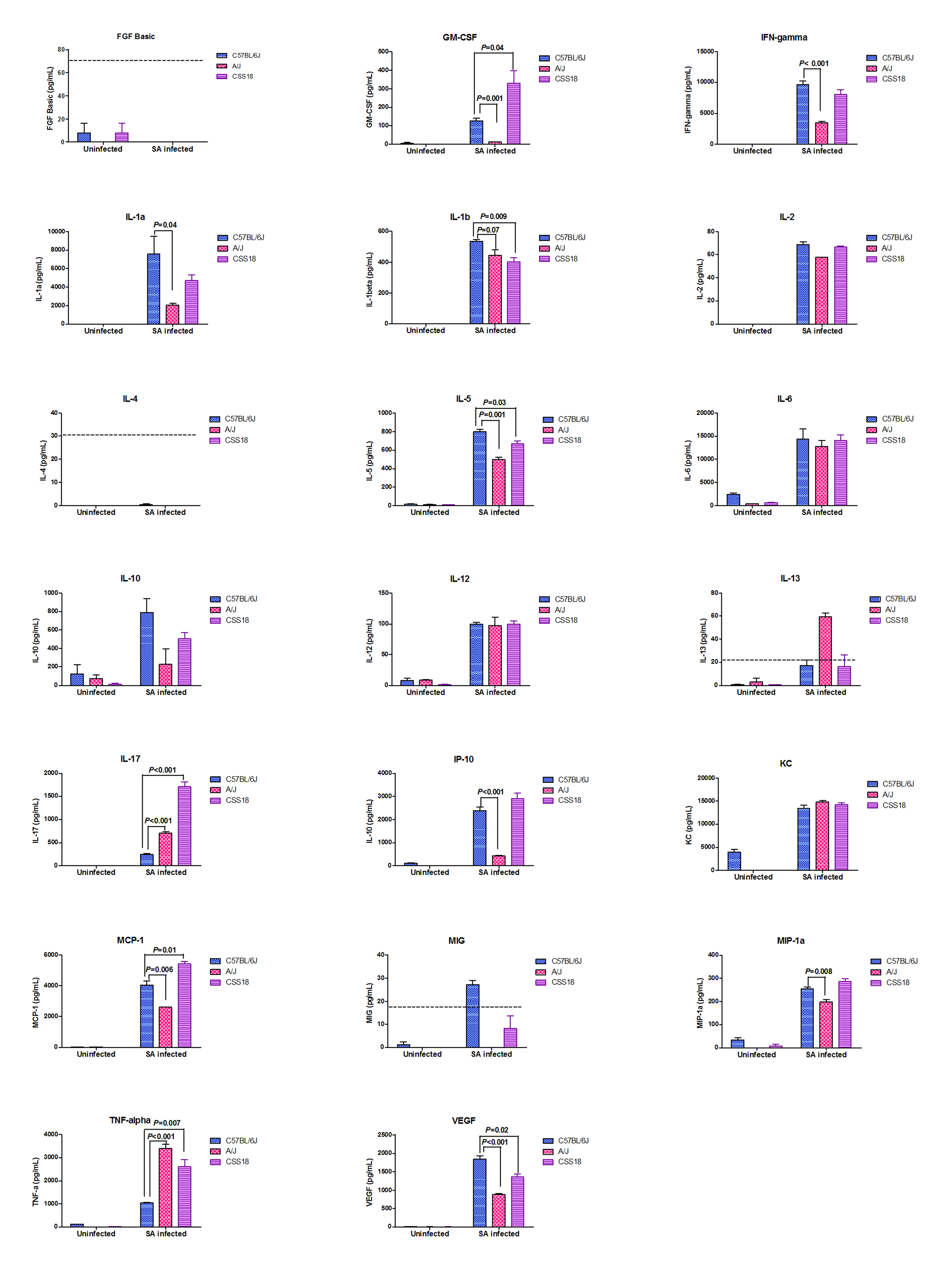

Supplement: Figure S6 — Cytokine/chemokine profiles of peritoneal macrophage from Chromosomal Substitution Strain 18 (CSS18) and C57BL/6J mice pre- and post- S. aureus stimulation. CSS18 mice, which contain A/J chromosome 18 but are otherwise genetically C57BL/6J, were used to minimize potential confounding effects of genetic susceptibility factors on A/J chromosomes 8 and 11. The peritoneal macrophage cells were isolated from C57BL/6J and CSS18 and cultured in RPMI1640 containing 10% FBS for 2 h. The remaining nonadherent cells were removed by washing with media and the macrophage monolayers were then stimulated to produce cytokine by incubation with 10 µg of S. aureus Bioparticles for 48 h at 37°C. The levels of cytokines/chemokines in cell culture supernatants at both pre- and post- stimulated conditions were measured by Luminex-based multiplex cytokine assay. The horizontal dashed lines indicate the minimum detectable concentration. Means and standard deviations of three independent experiments are shown. Values of P<0.05 were considered significant (unpaired two-tailed t-test). (3.52 MB TIF) [file ppat.1001088.s006.tif]

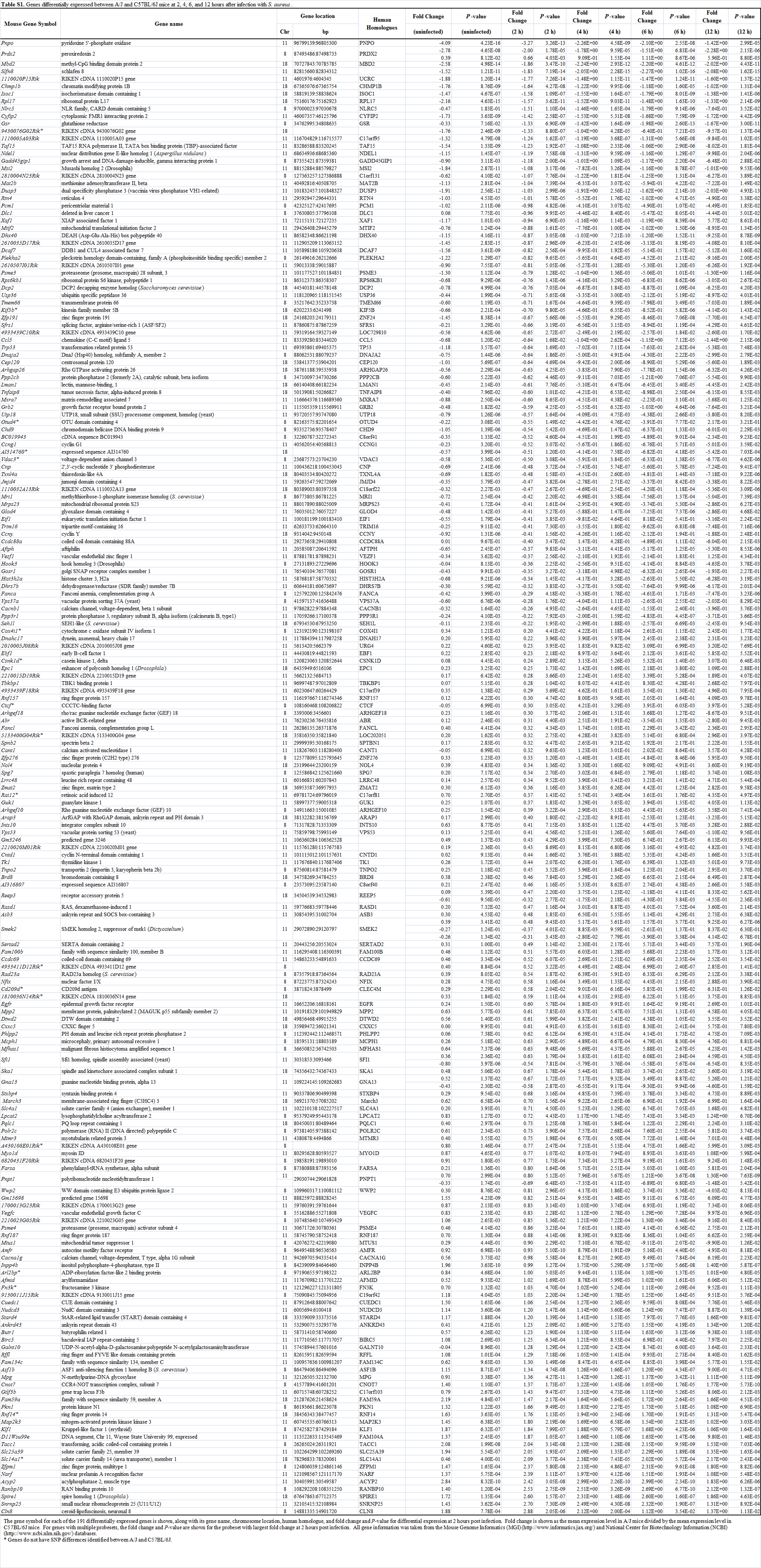

Supplement: Table S1 — Genes differentially expressed between A/J and C57BL/6J mice at 2, 4, 6, and 12 hours after infection with S. aureus. The gene symbol for each of the 191 differentially expressed genes is shown, along with its gene name, chromosome location, human homologue, and fold change and P-value for differential expression at 2 hours post infection. Fold change is shown as the mean expression level in A/J mice divided by the mean expression level in C57BL/6J mice. For genes with multiple probesets, the fold change and P-value are shown for the probeset with largest fold change at 2 hours post infection. All gene information was taken from the Mouse Genome Informatics (MGI) (http://www.informatics.jax.org/) and National Center for Biotechnology Information (NCBI) (http://www.ncbi.nlm.nih.gov/) databases. * Genes do not have SNP differences identified between A/J and C57BL/6J. (9.73 MB TIF) [file ppat.1001088.s007.tif]

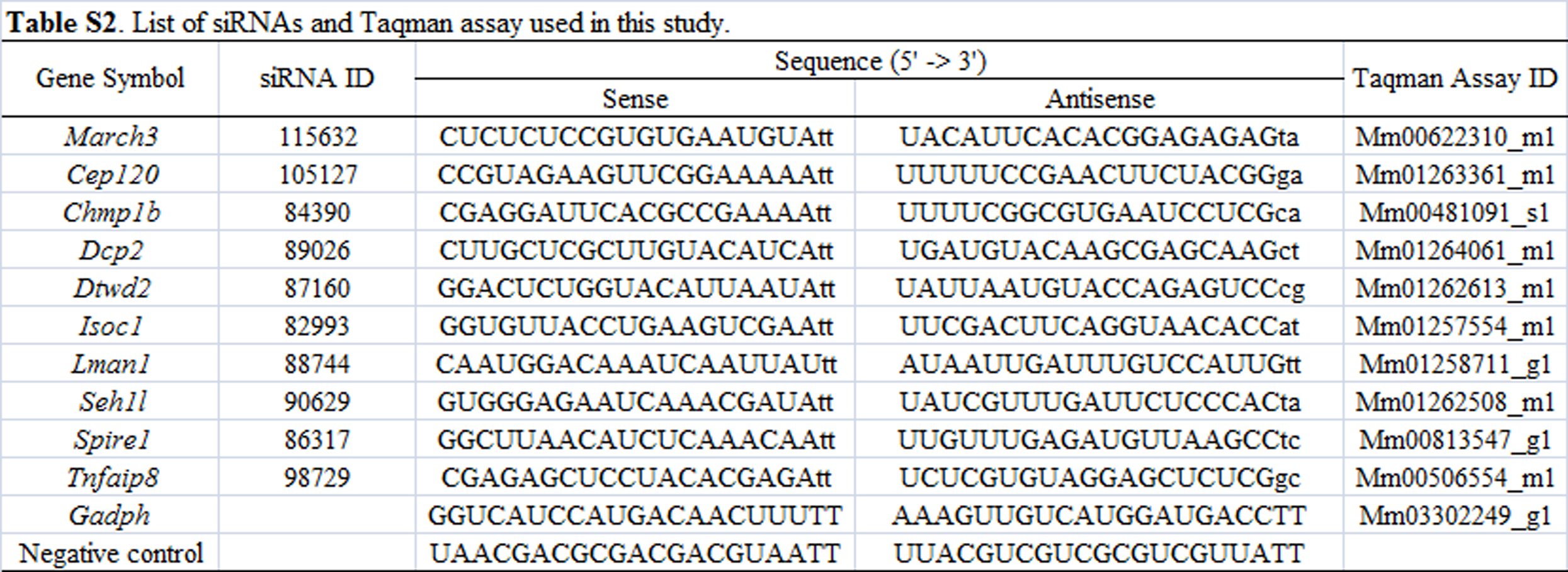

Supplement: Table S2 — List of siRNAs and Taqman assay used in this study. (2.08 MB TIF) [file ppat.1001088.s008.tif]
